# Fluid sources and overpressures within the central Cascadia Subduction Zone revealed by a warm, high-flux seafloor seep

**DOI:** 10.1126/sciadv.add6688

**Published:** 2023-01-25

**Authors:** Brendan T. Philip, Evan A. Solomon, Deborah S. Kelley, Anne M. Tréhu, Theresa L. Whorley, Emily Roland, Masako Tominaga, Robert W. Collier

**Affiliations:** ^1^ University of Maryland, College Park, MD, USA.; ^2^ University of Washington, Seattle, WA, USA.; ^3^ Oregon State University, Corvallis, OR, USA.; ^4^ Western Washington University, Bellingham, WA, USA.; ^5^ Woods Hole Oceanographic Institution, Woods Hole, MA, USA.

## Abstract

Pythia’s Oasis is a newly discovered seafloor seep on the Central Oregon segment of the Cascadia Subduction Zone, where focused venting emits highly altered fluids ~9°C above the background temperature. The seep fluid chemistry is unique for Cascadia and includes extreme enrichment of boron and lithium and depletion of chloride, potassium, and magnesium. We conclude that the fluids are sourced from pore water compaction and mineral dehydration reactions with minimum source temperatures of 150° to 250°C, placing the source at or near the plate boundary offshore Central Oregon. Estimated fluid flow rates of 10 to 30 cm s^−1^ are orders of magnitude higher than those estimated elsewhere along the margin and are likely driven by extreme overpressures along the plate boundary. Probable draining of the overpressured reservoir along the vertical Alvin Canyon Fault indicates the important role that such faults may play in the regulation of pore fluid pressure throughout the forearc in Central Cascadia.

## Teaser

Vigorous seepage offshore Oregon provides insight into the relationship between margin permeability and megathrust slip behavior.

## INTRODUCTION

It is well established that fluid-rock reactions, pore fluid pressure, and fluid flow have a profound effect on fault mechanics and the transition to seismogenic behavior within subduction zones (*1*–*4*). Pore fluid pressure along the megathrust in subduction zones is regulated by the balance between fluid sources (inputs) and fluid flow (outputs). Pore fluid overpressures develop when the rate of fluid input exceeds the rate of fluid drainage along fault zones and through the matrix sediments (*4*–*5*). Within the shallow forearc of subduction zones, the dominant sources of water contributing to the generation of pore fluid overpressure and fluid flow are (i) disequilibrium compaction within the accretionary wedge and underthrust sediments (*6*) and (ii) a series of mineral dehydration reactions that release bound water at progressively higher temperatures (*7*–*8*). Compaction is typically the primary source of water within the first 20 km from the trench where compressional forces drive the substantial reduction of sediment porosity (*3*, *5*, *6*). With increasing distance from the trench, the contribution of temperature-dependent mineral dehydration reactions increases. Total water input volumes are controlled by the composition of the incoming sediments and the thermal structure of the margin (*9*–*10*), with peak fluid production typically occurring within ~10 to 50 km of the trench.

Sediment permeability also plays an important role in governing the evolution of pore fluid pressure along the plate boundary given its control on the rate of fluid drainage. Within subduction zones, fault zones in the upper plate are important pathways for fluid flow and enhance drainage of the plate boundary as they often exhibit higher permeability than the matrix sediments (*4*, *11*). However, the disequilibrium compaction and the release of mineral-bound water in the outer forearc increases interstitial pore pressures, which counteracts the normal stress induced by the weight of the overlying sediment column (*12*), leading to a decrease in fault strength and locking along the plate boundary. At greater depths, the decrease in fluid production from compaction and dehydration reactions results in a decrease in pore fluid pressure and an increase in effective normal stress (*3*). The co-occurrence of diminishing temperature-dependent dehydration reactions, reductions in pore fluid overpressures, and the onset of seismogenesis in many subduction zones demonstrates that fluid production exerts a fundamental control on the buildup of interplate stress and is, therefore, a major topic of study (*2*, *9*, *13*–*15*).

Substantial uncertainties remain in the distribution of fluid production and associated excess pore pressures within subduction zones due to the challenge of making in situ observations of pore fluid pressure and fluid flow rates at depth. Long-range transport of chemically distinct fluids has been observed along the plate boundary and splay faults at several subduction zones through scientific drilling (*8*, *16*–*20*). However, only a few fault zones have been sampled, and direct measurements of the distribution and rates of fault-hosted fluid flow in the offshore forearc of subduction zones are sparse. For this reason, seafloor seeps have long been recognized as opportunities to study subseafloor fluid production because of their ubiquity across continental margins and their common connection to permeable fault systems that extend to the plate boundary and typically accommodate fluid flow rates of millimeters to centimeters per year (*2*, *4*, *8*, *15*, *21*–*22*). However, gaining insights into the initial chemical signatures of deep fluid production associated with fault zones is obscured because of (i) two-phase fluid flow related to the generation of methane in the shallow subsurface, (ii) solute diffusion, and (iii) in situ fluid-sediment reactions (*8*).

**Fig. 1.**
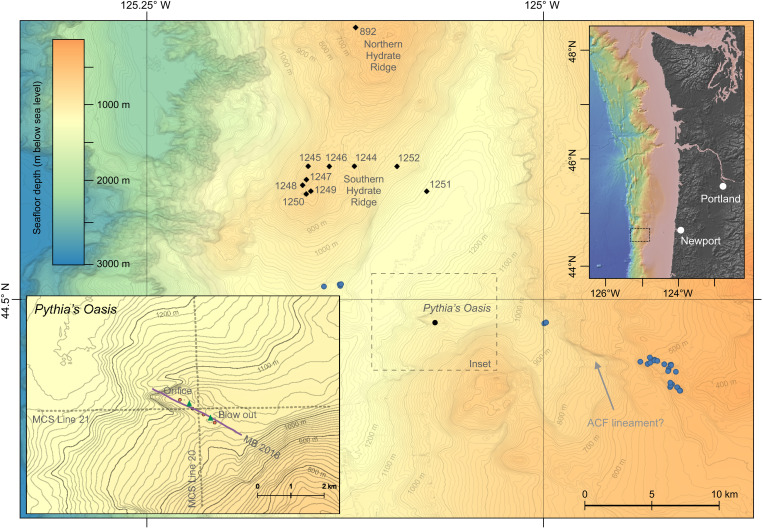
Map of Pythia’s Oasis and surrounding features. Pythia’s Oasis is located on the midcontinental slope ~80 km west of Newport, Oregon (see inset at top right). The seep site is located 10 km southeast of the well-studied Southern Hydrate Ridge, which was the subject of drilling during Ocean Drilling Program (ODP) Leg 204 (drill holes are shown as black diamonds); also shown is the location of ODP Hole 892 on Northern Hydrate Ridge, cored during ODP Leg 146. The multibeam-derived seep locations at Pythia’s Oasis are displayed as orange dots (see inset at bottom left), and the multibeam survey trackline is displayed in purple (labeled MB 2018). Also displayed are the 2015 locations of ROV-derived seafloor observations (green triangles; orifice and blowout) and the multichannel seismic (MCS) lines (dashed gray lines), and the locations of multibeam-derived seep locations during a survey along the Alvin Canyon Fault (ACF) in 2019 during expedition AT 42-17 onboard the R/V *Atlantis* (blue circles). The inferred location of the ACF (fig. S9) is supported by the lineament present in the bathymetry to the east of Pythia’s Oasis (gray arrow). The bathymetry shown is that of GMRT Version 3.7 (October 2019) (*82*), with contours drawn at 10-m (minor) and 100-m (major) depth intervals.

Here, we present the discovery of Pythia’s Oasis: a warm, high-discharge, water-dominated seafloor seep on the Central Oregon margin of the United States that allows direct access to deep-sourced fluids at the seafloor (Fig. 1). This seep was found during a routine transit onboard the R/V *Thomas G. Thompson* in 2014 and was first investigated using the remotely operated vehicle (ROV) *ROPOS* in 2015. Emitted at a rate and temperature not previously observed within a subduction zone context, the water discharged from this seep provides further insight into fluid sources at depth and offers a window into the permeability structure and distribution of overpressures within the forearc of the central Cascadia Subduction Zone (CSZ).

### Geologic setting

The CSZ, formed by the convergence of the Juan de Fuca and North American Plates (fig. S1), exhibits high temperatures along the plate boundary due to the young (5 to 9 Ma old) subducting lithosphere and the thick insulating sedimentary cover (*23*). The elevated temperature of the incoming sediments likely leads to the early onset of clay and opal dehydration reactions seaward of the deformation front (*8*, *10*) and to an up-dip shift in higher-temperature (e.g., 150° to 300°C) biotite-chloritoid-garnet schist dehydration reactions that normally occur much deeper within other subduction zones (*3*). This up-dip shift in the mineral dehydration sequence implies that shallower observations made within the CSZ can provide insights into processes in older, cooler subduction zones where these P-T conditions are typically out of reach.

Much like other subduction zones that exhibit variable locking characteristics along-strike (*24*–*25*), the distribution of offshore plate locking in the CSZ is heterogeneous (*26*–*27*). There are two distinct regions of high coupling in the northern and southern portions of the margin that exhibit strong offshore locking and a relatively narrow seismic-aseismic transition zone (*26*). In contrast, some models suggest that the central portion of the margin is defined by a narrow zone of locking near the deformation front, accompanied by a wide transition zone that extends inland within the central CSZ (*26*), which is potentially tied to high overpressures that result in underconsolidation relative to the northern portions of the CSZ (*28*). These overpressures may be the result of (i) the compaction of relatively high-porosity underthrust sediments compared with other sections of the CSZ (*28*), (ii) changes in permeability structure that restricts vertical fluid flow, and (iii) the contribution of a range of dehydration reactions that occur at both moderate and high temperatures. Pythia’s Oasis is located directly within this portion of the CSZ and therefore provides insight into overpressured conditions occurring at depth.

The Central CSZ has been a focus area for studies on forearc fluid sources and transport pathways with numerous seismic reflection surveys (fig. S2) (*29*–*31*) and two Ocean Drilling Program (ODP) legs (*32*–*33*). In addition, there have been several studies of seep systems across the central Oregon margin (*34*–*37*), which indicate a landward increase in dehydration-derived water emitted at seafloor seeps. Despite the intensity of sampling, little has yet been recovered that informs on fluid-rock reactions occurring from 150° to 300°C, which is typically coincident with the onset of seismicity (*3*). In this context, Pythia’s Oasis stands out for not only being a water-dominated system but also for potentially informing on reactions occurring within the seismogenic zone in the Central CSZ.

## RESULTS

### Bathymetry and water column observations

**Fig. 2.**
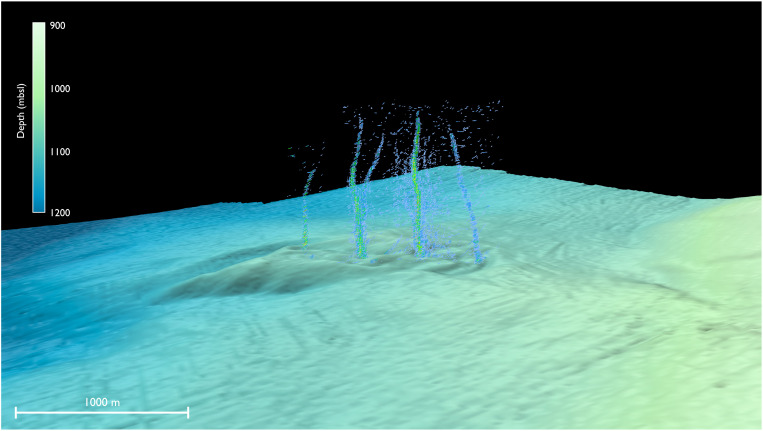
Sonar image of bubble plumes at Pythia’s Oasis. This is a perspective view looking northeast at the bubble plumes rising above Pythia’s Oasis, imaged during a 2018 transect onboard the R/V *Revelle* using a Kongsberg EM122 multibeam sonar. The hydroacoustic data were filtered using Fledermaus Midwater and reveal gas bubbles rising >500 m into the overlying ocean (image is 2× vertically exaggerated). The bathymetry shown here was collected onboard the R/V *Thompson* in 2015 and has been gridded at 20-m horizontal resolution.

The active seep area at Pythia’s Oasis is defined by a ~1.5-km-long sedimented promontory at 1040 m below sea level (mbsl), bounded to the north by a 15° slope that terminates ~85 m below (Fig. 1). To the south, bubble emissions are confined by a >75-m-across and 10-m-deep semicircular moat. Initial multibeam surveys in 2014 and 2015 (table S1) indicated two distinct vents spaced ~380 m apart, with bubble plumes at both locations rising 575 m into the overlying ocean. Follow-on hydroacoustic surveys in 2016, 2017, and 2018 (Fig. 2) imaged two additional vent locations: one at the northern end of the promontory and the other originating from within the moat at the southern end of the field. The acoustically derived vent locations are spaced at ~380-m intervals on a 1-km-long southeast (SE)–northwest (NW) trending line along the length of the promontory. Two or more vents were actively emitting gas on each of the eight acoustic surveys conducted over the 5-year period.

### Seafloor observations

**Fig. 3.**
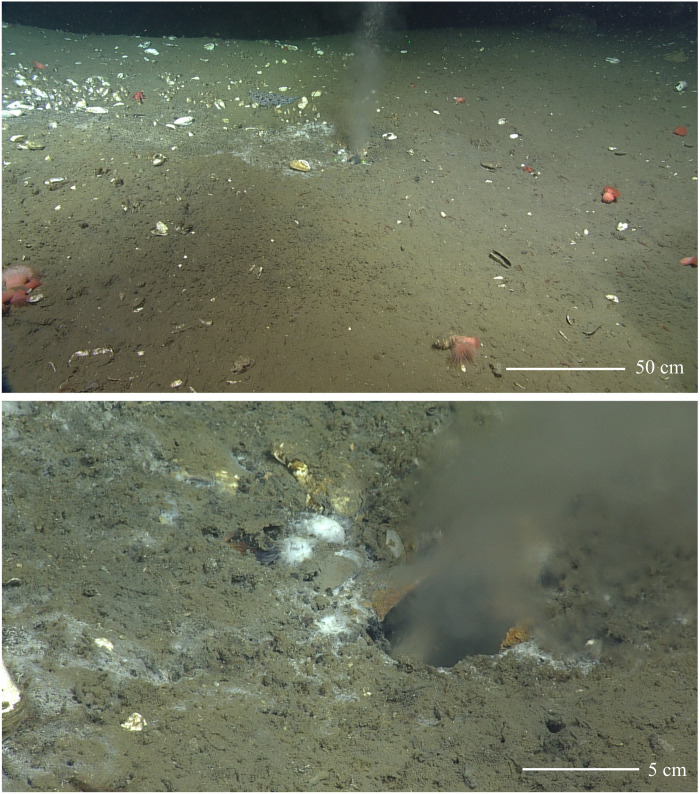
The warm, water-dominated vent at Pythia’s Oasis. The high-volume, 12°C flow at Pythia’s Oasis is confined to a 5-cm-diameter vent located ~380 m away from the collapse zone. These images were taken during *ROPOS* dive R1858 on 24 July 2015. The focused discharge of fluids from this vent has been observed during multiple ROV dives in 2015 and 2016.

The seafloor at this site was first visually surveyed in 2015 (during the *ROPOS* ROV dive R1858 on the R/V *Revelle*), when a steady stream of bubbles was traced to a well-defined seafloor orifice 5 cm in diameter with authigenic mineral crusts lining the interior of the opening (movie S1). Particle-laden water with a refractive index different from that of background bottom water was seen buoyantly rising out of the orifice at visibly elevated flow rates relative to the background flow field (Fig. 3). During these initial observations, the onboard conductivity-temperature-depth (CTD) detected a 0.6 practical salinity unit (PSU) salinity drop at a distance several meters from the orifice (fig. S3), indicating that low-salinity water was being emitted at considerable flow rates to alter seawater salinity in the vicinity of the vent. In addition, bottom water temperatures near the vent were 0.1°C warmer than background values, reflecting the discharge of water with an elevated thermal signature.

On the basis of video collected during the 2015 dive, linear rates of water flow are estimated to be between 10 and 30 cm s^−1^, which is equivalent to volume fluxes of 196 to 589 ml s^−1^. Temperature measurements made within the orifice during follow on dives with the ROV *Jason* were 11.8°C (2016) and 12.6°C (2017); both measurements were substantially elevated above background temperatures of ~3°C and confirm the unique discharge of warm water from the orifice at Pythia’s Oasis.

During the 2015 dive, the southernmost bubble plumes were traced to a 10-m-deep, circular collapse zone ~50 m in diameter and located ~380 m southeast of the water-dominated seafloor orifice (Fig. 1). This zone is characterized by extremely hummocky terrain covered in bacterial mats, intermittent pock marks and crevasses, and a rim covered in filamentous bacteria (movie S1). Bubble emissions within the collapse zone were traced to seafloor vents that were coincident with the release of shimmering, diffuse fluid emanating from the sediments. While no temperature measurements or water samples were collected at this location, the release of water with an anomalous refractive index likely reflects the emission of low-salinity or moderate-temperature fluids, or both, in this region.

### Orifice chemistry

Push cores collected within 1 m of the orifice using the ROV *Jason* in 2016 indicate seawater recharge: the concave-up curvature of most profiles is a strong indicator of the downward advection of seawater near the orifice (fig. S4). The near-complete consumption of sulfate below 12-cm depth in both cores places the sulfate-methane transition zone (SMTZ) at that depth; below this depth, sulfate is completely depleted in pore waters. Thus, sulfate can be used to correct for mixing between seawater and the discrete vent samples collected via ROV *Jason*. Assuming a fluid end-member that contains zero sulfate (i.e., sourced from below the SMTZ) and a seawater sulfate end-member concentration of 28.9 mM, the sulfate concentration measured within the orifice fluids (5.78 mM) represents a mix of 20% entrained seawater and 80% sulfate-free end-member fluid. All samples collected using Major samplers in 2016 were corrected in this manner. Samples collected using isobaric gas-tight (IGT) samplers in 2017 were not preserved with zinc acetate for sulfate measurements and thus are uncorrected for seawater mixing.

Fluids sampled from within the orifice using the Major samplers were depleted in deuterium (−10.4‰ δD) and moderately depleted in ^18^O (−0.1‰ δ^18^O; table S2). Mixing-corrected values of the major seawater constituents reveal a calcium-rich end-member (53.4 mM Ca), with depleted concentrations of chloride (347 mM), sodium (245 mM), magnesium (5.1 mM), and potassium (2.1 mM) with respect to seawater. Uncorrected IGT values display similar trends, with a mean difference between sample collection devices of 6 to 40%. The IGT samples are depleted in deuterium (−8.8‰ δD) and slightly enriched in ^18^O (0.5‰ δ^18^O). The fluids are enriched in calcium at 50.4 mM and depleted in sodium (209.8 mM), magnesium (7.3 mM), and potassium (2.2 mM); all values reported here for IGT samples represent the mean of two duplicate samples. The IGT samples are extremely enriched in the minor elements boron (6026.4 μM) and lithium (275.5 μM): ~15 times that of their concentrations in seawater (table S2).

Assuming that chloride behaves conservatively within the temperature range of the fluid source(s) at Pythia’s Oasis, the addition of fresh water due to mineral dehydration reactions at depth has diluted chloride concentrations in the vent fluid to 62% of that of seawater. If dilution is the only process affecting the other solute concentrations, then all solutes would have concentrations that are 62% of their values in seawater. However, the concentrations of sodium (51%), magnesium (9%), and potassium (20%) with respect to seawater are evidence of reactions both at the source and along the flow path and are an indication that there is considerable K and Mg consumption through this process.

Note that Cl has been shown to be incorporated into authigenic hydrous minerals at temperatures ≥250°C (*38*–*40*). Thus, if a major fraction of the fluid feeding the Pythia’s Oasis vent is sourced at these temperatures, then the estimated amount of dilution by fresh water would be less and the amount of uptake of K and Mg along the flow path would be greater.

### Subseafloor structure and heat flow

High-resolution seismic lines collected over Pythia’s Oasis in 2017 imaged down to 0.3-s two-way travel time (TWT) beneath the seafloor or ~225 m below the seafloor (mbsf). The high-resolution lines show the relationship between deformed sediments of the accretionary complex, which overlie slope basin sediments, and the base of the gas hydrate stability zone indicated by the presence of a bottom-simulating reflector (BSR). The BSR, which is interpreted to represent the phase boundary between free gas and gas hydrate when the amount of gas exceeds its solubility in the pore water, is a proxy for subsurface temperature and is widely used to infer local variations in heat flow at continental margins (*41*–*42*). Simple one-dimensional steady state models of upward flow of warm fluid indicate that BSR perturbations are sensitive to flow rates in the range of ~0.1 to 10 cm year^−1^, with lower rates having no detectable effect and faster rates resulting in the BSR shoaling toward the seafloor beneath a vent (*43*–*44*). Faster flow rates may occur intermittently but cannot be resolved from BSR observations alone.

**Fig. 4.**
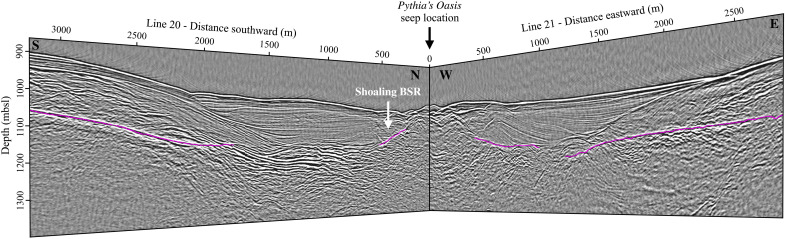
Subseafloor structure beneath Pythia’s Oasis. This is a perspective view looking northwest at the intersection of seismic lines 20 (left) and 21 (right); for line orientation, see Fig. 1. Pythia’s Oasis is centered at the origin of this plot (see black arrow), and the BSRs are highlighted in pink. The seep is positioned atop a conical contact between underlying accreted sediments and continental slope sediment infill that is approximated by the sloping BSRs near the seep. BSR shoaling is indicated as a white arrow.

The north-south transect [multichannel seismic (MCS) Line 20] acquired during the RR1718 cruise on R/V *Revelle* in 2017 reveals that the seep is positioned atop a conical contact between underlying accreted sediments and continental slope sediment infill (Fig. 4). Directly south of the seep lies a ~140-m-thick basin overlying accreted sediments with evidence of slumping near the seafloor; this slump is evident in the multibeam bathymetry and measures ~700 m in diameter (Fig. 1). Underlaying the basin is a region of high reflectivity through which the regional BSR is lost. Given that the clear contact between accreted and slope sediments is nearly continuous along the length of the line, this high-reflectivity patch likely represents discrete fluid–charged horizons within the accreted sediment. Further to the north are the BSR shoals from 130 to 51 mbsf before disappearing 240 m south of the seep. North of the seep, the BSR deepens within the accreted sediments and beneath subhorizontal layered sediments, which are likely due to a combination of uplift and onlapping within the basin to the north (fig. S5).

The east-west transect (MCS Line 21) images a 105-m-thick basin to the east of the seep (Fig. 4), a continuation of the basin imaged to the south of the seep in Line 20. This portion of the basin contains slope sediments overlying older accreted sediments, and it is crosscut by a westward shoaling BSR. The subhorizontal layering observed in the eastern basin sediments is interpreted as an indication of uplift centered around the seep. To the west of the seep, along MCS Line 21 (fig. S6), an eastward-shoaling BSR is located well within the underlying accretionary complex and disappears at a depth of 174 mbsf, 650 m west of the seep.

**Fig. 5.**
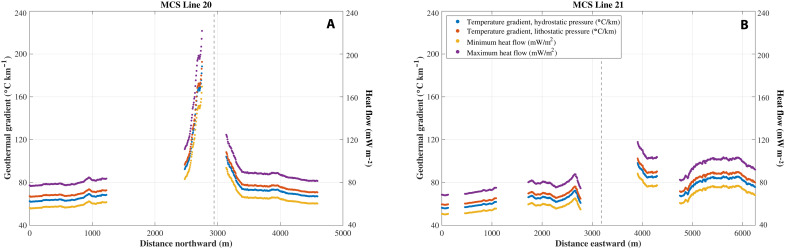
Geothermal gradient and heat flow plots. Geothermal gradient and heat flow calculated from the observed seafloor and BSR depth along MCS lines 20 (**A**) and 21 (**B**). The in situ pressure at the BSR is likely somewhere between hydrostatic and lithostatic and may vary along the profile; therefore, the temperature gradient is shown assuming either hydrostatic or lithostatic pressure at the BSR to calculate the temperature corresponding to the gas hydrate stability boundary. Heat flow bounds were calculated assuming a thermal conductivity of 0.9 W m^−1^ K^−1^ and hydrostatic pressure to estimate the minimum heat flow and a thermal conductivity of 1.15 W m^−1^ K^−1^ and lithostatic pressure to estimate the maximum heat flow. The position of the seafloor seep is indicated in each plot with a vertical, dashed line.

Geothermal gradients calculated using BSR picks are 62° to 73°C km^−1^ at background sites along the north-south transect (MCS Line 20) and increase to 92° to 193°C km^−1^ within 500 m of the seep (Fig. 5A). These geothermal gradients correspond to near-seep heat flow values of 83 to 222 mW m^−2^. Background geothermal gradients in the east-west transect (MCS Line 21) are generally lower to the west (56° to 65°C km^−1^) than to the east (67° to 90°C m^−1^). Within ~1 km of the seep, geothermal gradients increase to 61° to 103°C km^−1^ (Fig. 5B), corresponding to heat flow values of 55 to 118 mW m^−2^. The background geothermal gradients near Pythia’s Oasis are consistent with background values of ~55°C km^−1^ measured elsewhere throughout the Hydrate Ridge region (*29*, *45*).

## DISCUSSION

The fluid flow rates inferred from analyses of Pythia’s Oasis dive video are the highest seafloor rates yet measured within the CSZ. Previous seafloor measurements made using benthic barrel fluid flowmeters (*34*, *46*) along the first anticlinal ridge offshore Oregon, detected linear flow rates of 10^3^ to 10^4^ cm year^−1^, while similar studies at Hydrate Ridge (10 km to the northwest of Pythia’s Oasis) (*46*–*49*) reported maximum flow rates between 10^1^ and 10^5^ cm year^−1^. Each of these values are in sharp contrast to the estimated 3 to 9 × 10^8^ cm year^−1^ linear flow rates within the orifice at Pythia’s Oasis, which correspond to annual water fluxes of 6 to 18 × 10^3^ m^3^ year^−1^. The total water output at Pythia’s Oasis is likely greater than these estimated fluxes given the visual evidence of fluid release elsewhere at the site, particularly within the collapse zone. In addition, the elevated geothermal gradients of 83° to 222°C km^−1^ near Pythia’s Oasis stand in sharp contrast to the relatively narrow range of values of ~55°C km^−1^ measured throughout much of the Hydrate Ridge region (*29*, *45*), indicating the presence of strong vertical heat transport at Pythia’s Oasis.

Intense bubble plumes were imaged emanating from this site in all eight surveys from 2014 to 2018 (table S2), and bubble plumes have been subsequently documented annually by hull-mounted sonar up to 2022 during Ocean Observatory Initiative Regional Cabled Array expeditions. The seismic reflection data indicating shoaling of the BSR above background values within 500 m of the seep confirm that upward aqueous flow (and thus high heat flow) is occurring over a broad region beneath Pythia’s Oasis (Fig. 5). This is in contrast to Southern Hydrate Ridge, where flow has been shown to be gas-dominated (*33*, *50*–*52*) and where there is little elevation in the apparent heat flow near the summit (*45*). At Pythia’s Oasis, where the nearest detectable BSR to the orifice is roughly 150 m away and is 50 m shallower than the background BSR depth, a first-order estimate for the time required to shift the lower limit of gas hydrate stability indicates that elevated flow rates are sustained on the order of ~1500 years (see Methods and fig. S7). Given the time required to perturb the BSR, Pythia’s Oasis must be a long-lived seep. The question remains whether the measured rates of water discharge are compatible with existing knowledge of fluid sources within the CSZ.

On the basis of the geochemical data, three hypotheses are presented below for the source of water discharged at Pythia’s Oasis. These sources include accreted sediments, under thrust sediments, or both. Comparison of water discharge rates with exiting fluid budgets within the central CSZ demonstrate that discharge must either be transient or is the result of focusing over a broad region where fluids are channeled into a high-permeability conduit.

### Water sources and fluid budgets

End-member pore water sampled from 86 to 467 mbsf in the central CSZ during ODP Legs 146 (*32*) and 204 (*33*) indicate a progressively larger component of dehydration-derived fluids with distance from the deformation front, achieving a minimum pore water chloride value (432 mM) 19 km from the toe. Fluids sampled from the orifice at Pythia’s Oasis, at 20 km from the trench, are consistent with a progressive freshening of seep fluids with distance landward of the deformation front (fig. S8). While the 346 mM Cl^−^ measured at Pythia’s Oasis is consistent with this trend, all other measured constituents represent substantial outliers. Relative to nearby borehole measurements at the summit of Southern Hydrate Ridge (ODP Sites 1249 and 1250) (*33*), the minor elements (B and Li) are 4 to 10× enriched, and the major elements are 2 to 9× depleted, with the exception of Ca that is 12 to 17× enriched.

The chemical composition of fluids collected from the orifice at Pythia’s Oasis indicates that they represent a mixture derived from sediment compaction and mineral dehydration reactions. Assuming that the only source of low-chloride water is dehydration reactions (a realistic assumption given that δ^18^O and δD do not trend toward gas hydrate dissociation in Pythia’s Oasis samples) and that chloride concentrations of compaction-derived fluids closely resemble that of seawater (559 mM Cl^−^) (*50*), the 346 mM Cl^−^ measured in the Major sample represents a mix of 38% dehydration and 62% compaction sources. The approximately two-thirds contribution of compaction-derived fluids to the vent corresponds to an annual volume of 4 × 10^3^ to 11 × 10^3^ m^3^. This is likely an underestimate of the total volume of compaction-derived fluid flow at Pythia’s Oasis given the three additional documented sites of discharge at the seafloor (Figs. 1 and 2).

In the central CSZ, pore water inputs at the deformation front are sourced from the 3-km-thick incoming sediment section that overlies the Juan de Fuca plate, with roughly equal partitioning between accreted and subducted sediments (*28*). Pore water volume fluxes have been estimated for these reservoirs using seismic reflection data, velocity-porosity relationships (*28*), and a convergence rate of ~35 km Ma^−1^ (*53*). Sediment porosities (*n*) within the accreted section at the deformation front (*n* = 0.27) correspond to a pore water flux of 14 × 10^3^ m^3^ year^−1^ per kilometer of trench length, while the underthrust sediments (*n* = 0.15) account for a flux of 8 × 10^3^ m^3^ year^−1^ per kilometer of trench length. The compaction-derived water discharge rates estimated for the 5-cm-diameter orifice at Pythia’s Oasis (4 × 10^3^ to 11 × 10^3^ m^3^ year^−1^), therefore, account for 29 to 79% of the accreted pore water flux, 50 to 100% of the underthrust flux, and 18 to 50% of the total annual pore water flux into the subduction zone per kilometer of trench length in the central CSZ.

The remaining approximately one-third of fluid emitted at Pythia’s Oasis is likely sourced from dehydration reactions and associated freshwater production occurring under thermal conditions that meet, and possibly exceed, peak smectite-illite (S-I) transformation (60° to 150°C) (*8*). One indicator of in situ temperature is boron, which is desorbed from clay surfaces at temperatures of ~150°C (*54*–*55*). At Pythia’s Oasis, boron concentrations (6000 μM) are extremely enriched with respect to typical seawater values (420 μM). In addition, Pythia’s Oasis boron concentrations are in excess of those produced by hydrothermal experiments conducted between 50° and 350°C on terrigenous sediments from the incoming Juan de Fuca Plate (*55*) and hemipelagic muds (*54*). Furthermore, lithium (another tracer of deep-sourced fluids) was measured at concentrations (~275 μM), consistent with those produced by terrigenous sediments from the Juan de Fuca Plate at temperatures in excess of 200° to 250°C (*55*).

**Fig. 6.**
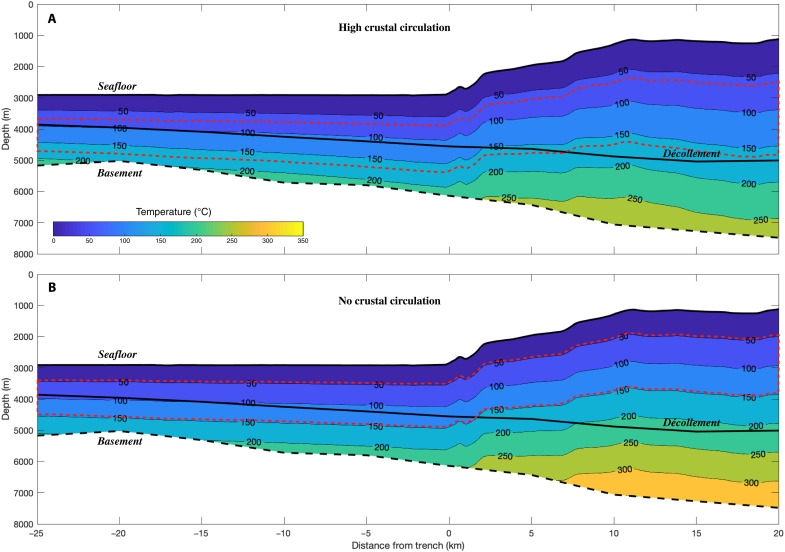
Subseafloor temperature structure seaward of Pythia’s Oasis. Cross-sectional view of the sedimentary temperature distribution perpendicular to the deformation front in Central Oregon. Shown are two end-member thermal models (*23*) that constrain (**A**) the minimum thermal gradient derived by permitting high fluid circulation within the oceanic crust and (**B**) the maximum thermal gradient based on the condition of no crustal fluid circulation. The horizons are derived from MSC data (*28*): Shown are the basement (dashed black line) and décollement (solid black line). Seaward of the trench (indicated by negative distances), the solid black line represents a distinct stratigraphic boundary identified in MSC data (*28*). The region of peak water production from smectite dehydration (50° to 160°C) is bounded by dashed red lines. The conditions for peak dehydration landward of the deformation front are completely confined to the accreted sediments. Pythia’s Oasis is located at the right edge of the image, approximately 20 km landward of the trench.

Taken collectively, B and Li enrichments and depletion in Cl and K indicate a minimum source temperature of 150° to 250°C, which approaches the upper limit of smectite dehydration. To estimate the depth of this temperature range within the central CSZ, thermal models developed for Cascadia (*23*), coupled with updated estimates of wedge and underthrust geometry (*28*), were used. This revised perspective (Fig. 6) indicates that (i) there is ongoing smectite dehydration producing water within the accretionary wedge due to relatively cool accretion followed by wedge thickening and (ii) that the remaining contribution is from fluids sourced at temperatures exceeding the S-I transition (>250°C), which would need to originate from the plate boundary or underthrust sediments. The presence of a broad and landward-thickening zone within the wedge, which is ideal for peak S-I dehydration, represents an underappreciated contribution of fresh water to the Cascadia hydrologic system.

Within the Central CSZ, incoming sediments subducted beneath the plate boundary have already undergone smectite dehydration seaward of the deformation front (Fig. 6). In addition, this perspective indicates that there is continued production of fresh water from the S-I transition within the accretionary prism, which is supported by a smectite concentration of ~29 weight % within the accreted sediments (*56*). This source of freshwater production is not considered in existing models of subduction zone hydrogeology, which only track dehydration reactions within the underthrust sediments. In summary, the accreted sediments largely do not experience temperatures warm enough to initiate the S-I transition seaward of the deformation front, and the zone of peak smectite dehydration (50° to 160°C) steps up to the décollement within 2 km landward of the deformation front and remains exclusively within the wedge, thereafter, increasing in thickness with distance eastward (Fig. 6).

It is unlikely that discharge at Pythia’s Oasis is sourced from pore water entering only in a narrow section of the subduction zone given the well-documented ventilation occurring within the wedge seaward of this seep. Numerous geophysical surveys have confirmed the presence of thrust faults between the deformation front and Pythia’s Oasis (*29*, *35*, *57*–*59*), and fluid samples collected from seafloor seeps along the first and second anticlinal ridges indicate that fault-controlled seepage is tapping compaction-derived fluids from within the wedge (*60*–*61*). Furthermore, the overall reduction in pore water volume within the accretionary wedge is evidenced by a decrease in average porosity from 0.27 at the deformation front to 0.20 within 20 km landward (*27*), thereby diminishing the pore water volume available to drive flow at Pythia’s Oasis. Sustaining elevated water discharge rates may, therefore, require the integration of flow from a broad section of the margin, a process examined in the following section.

### Fluid transport pathways and overpressured reservoirs

One likely mechanism for integrating flow laterally in the central CSZ is the oblique strike-slip faults that extend from the abyssal plain to the upper continental slope-outer shelf region (fig. S9). These near-vertical faults extend throughout the entire sediment section on the abyssal plain (*58*, *62*) and likely propagate through the oceanic crust on the incoming plate (*63*). Numerous NW-SE trending features have been noted along the margin and interpreted as a series of “bookshelf” strike-slip faults accommodating compression (fig. S9) (*58*, *64*). Landward of the deformation front, the strike-slip faults are thought to intersect the décollement (*65*) and may reach the subducting oceanic crust (*66*), indicating a viable high-permeability pathway for the deep-sourced (>2 km) fluids. The colocation of vigorous seepage along the Wecoma strike-slip fault (located 60 km north of Pythia’s Oasis) confirms that strike-slip faults can host high rates of fluid transport within the region (*65*), and the alignment of the Alvin Canyon Fault (ACF) with Pythia’s Oasis (Fig. 1 and fig. S9) is further evidence of a vertical transport pathway facilitating rapid fluid flow.

**Fig. 7.**
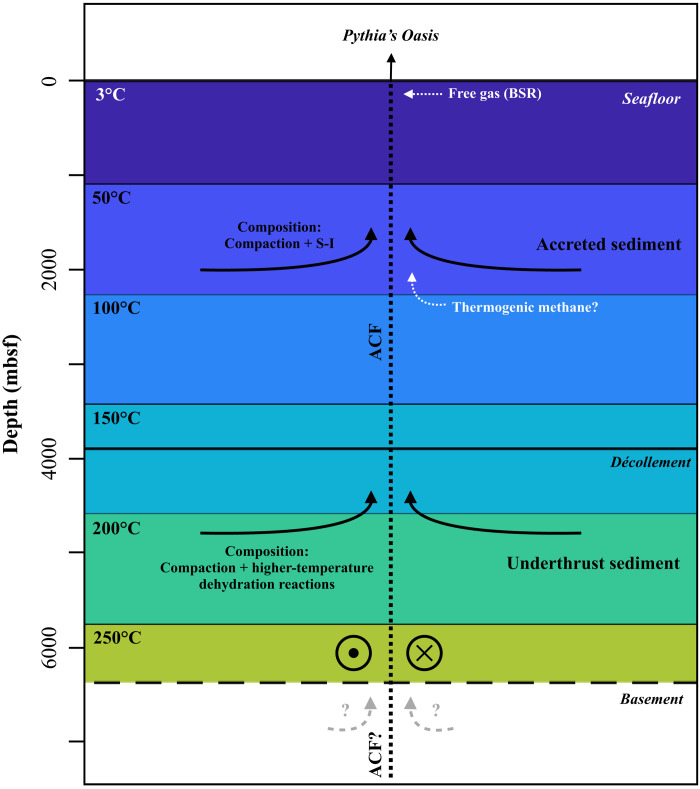
Schematic of possible flow paths and fluid sources beneath Pythia’s Oasis. The potential colocation of this seep and the Alvin Canyon strike-slip fault (ACF, vertical dashed line) may allow for the integration of fluids from the accreted and underthrust sediments and potentially from the oceanic basement. Although discharge at Pythia’s Oasis is water-dominated, the presence of gas is well documented and could either be microbial or thermogenic in origin; the depths of gas formation are labeled in white, with the microbial component being sourced from at or near the BSR. The depths of the décollement (thick horizontal line) and basement (dashed horizontal line) are the same as those presented in Fig. 6, 20 km landward of the deformation front (*28*). The color scale is that of the high crustal circulation model (*23*).

Although poorly defined in the vicinity of Pythia’s Oasis, the ACF is thought to have a variable width of 1 to 6 km along its 70-km length (*64*) and may be manifested in the seafloor morphology as a bathymetric lineament 4 to 5 km east of Pythia’s Oasis (Fig. 1). This interpretation of the ACF as a possible transport pathway for fluid flow occurring at Pythia’s Oasis is further supported by (i) the orientation of the lineament ~250 m to the southwest of the vent (Fig. 1) and (ii) the linear orientation of the observed seeps (Figs. 1 and 2), which indicates fault control and aligns with the strike direction of the ACF. The ACF is also thought to underlie Southern Hydrate Ridge (*64*), 10 km northwest of Pythia’s Oasis. In addition, numerous seafloor seeps were detected elsewhere along the projection of the ACF in the vicinity of Pythia’s Oasis during a 2019 expedition (Fig. 1 and fig. S9). Therefore, the ACF may represent a pathway for channeling flow from a broad lateral and vertical swath of the wedge and possibly from the plate boundary and underthrust sediments (Fig. 7). An inferred thrust fault beneath Pythia’s Oasis (*67*) may constitute a secondary transport pathway that intersects the ACF at depth, although there is inconclusive evidence of such a thrust fault based on more recent deep seismic reflection data in the area (figs. S1 and S2).

Despite the probable colocation of Pythia’s Oasis and the high-permeability ACF, it is highly unlikely that the extreme flow rates measured at the seafloor (10 to 30 cm s^−1^) are sustained at depth given the unrealistic pore pressures and permeabilities that would be required to support such flow. Assuming a realistic fault zone permeability of 10^−14^ m^2^ (*3*, *11*), Darcy’s law predicts that fluids with a viscosity of 10^−3^ kg m^−1^ s^−1^ and a fluid density of 1000 kg m^−3^ would require an impractical pore pressure (λ^*^ = 10^5^, where λ^*^ is the modified pore pressure ratio) to drive flow at the observed rates. Conversely, at a more reasonable (but still high) pore pressure of λ^*^ = 0.9 (representing near-lithostatic conditions), the required permeability (10^−9^ m^2^) far exceeds those typical of marine sedimentary environments and is several orders of magnitude greater than typical oceanic crust permeabilities (*68*).

Under more realistic permeability (10^−14^ m^2^) and pore pressure (λ^*^ = 0.9) conditions, Darcy’s law holds that flow should not exceed 4.7 m year^−1^. If confined to a fault zone of 10-m width, then flow at 4.7 m year^−1^ would need to be distributed over a lateral distance of 130 to 390 m along the fault to supply enough water to the seafloor to account for discharge rates of 10 to 30 cm s^−1^ at the orifice. Although the pore pressure ratio required to drive flow under these conditions scales inversely with the width of the conduit, any scenario for concentrating extreme volumes of water would require either (i) a high pore pressure ratio at the fluid source assuming permeabilities of 10^−14^ m^2^ or (ii) permeabilities even greater than 10^−14^ m^2^ assuming moderate pore pressures (λ^*^ ≤ 0.7) (*4*).

Assuming that water discharged at the orifice is transported from depth over a broad zone at realistic flow rates (not exceeding 4.7 m year^−1^), then this flow must be concentrated near the surface in zones of elevated permeability such that discharge match the estimated 10 to 30 cm s^−1^. The likely colocation of Pythia’s Oasis with the ACF provides a scenario under which fractures can form at low pressures near the seafloor. Because the pore pressure required to initiate hydrofracturing within faults is largely dependent on the magnitude of the least principal stress (*69*), pore pressures must exceed lithostatic load to overcome the vertical orientation of the least principal stress within thrust faults. In contrast, the horizontal principal stress orientation in strike-slip faults may allow hydrofracturing to occur at lower pore pressures within the upper 100 m (e.g., λ = 0.8) compared to the lithostatic pore pressures (λ = 1.0) required to hydrofracture a thrust fault at similar depths (*69*). The elevated overpressures driving flow beneath Pythia’s Oasis are likely sufficient to induce hydrofracturing within the ACF, either at depth or near the seafloor—a process capable of concentrating flow from across the fault zone to a well-defined, focused outlet at the seafloor.

### Implications for subduction zone hydrogeologic models

The conclusions that Pythia’s Oasis fluids are likely sourced from at or near the plate boundary and that an overpressured reservoir is required to drive the observed flow suggest that high overpressures (e.g., λ^*^ = 0.9) are present along the décollement in the central CSZ. The inferred high overpressures along the plate boundary are consistent with seismic reflection data indicating underconsolidation of the underthrust sediments offshore central CSZ (*28*) and low coupling along the plate boundary inferred from GPS data (*26*).

Previous surveys of in situ pore pressure in this region reveal overpressures reaching >85% of lithostatic load (*69*) within a zone of confined fluid flow (*8*) at the frontal thrust near the deformation front. Our results indicate that elevated pore pressures persist well beyond the frontal thrust in the central CSZ. Such an overpressured region is likely to have implications for the distribution of effective normal stress either within the accretionary wedge, along the décollement, or within the underthrust sediments. If fluids sampled at Pythia’s Oasis are indeed sourced from the décollement, then it is likely that effective stress is low and may be one of the mechanisms contributing to partial locking within this section of the CSZ (*26*, *28*).

The presence of strike-slip faults within central CSZ likely provide efficient, vertical pathways that drain overpressures generated within the forearc through a combination of compaction and mineral dehydration reactions. Given that hydrofracturing can occur at relatively low pore pressures within strike-slip faults (*65*) and that the ACF is inferred to intersect the plate boundary, near-lithostatic pore pressures along the plate boundary would be sufficient to drive fracture permeability and the elevated flow rates observed at Pythia’s Oasis. While this mechanism would eventually alleviate local fluid pressures along the décollement, fluids could be sourced from anywhere that is overpressured within the system (across and along strike), potentially enabling flow for sustained periods. The BSR anomalies that indicate sustained flow for ~1500 years at Pythia’s Oasis support this interpretation.

The presence of both compaction- and dehydration-derived fluids in samples collected at Pythia’s Oasis indicate that flow is likely integrated from multiple depths, which may include overpressure development within the accretionary prism from both compaction and the S-I transition, as well as from the underthrust sediments (Fig. 7). In addition, while the intermittent observations of a dynamic system demonstrated by our study make it difficult to determine long-term variations in flow rates and chemistry with certainty, the available evidence (including authigenic mineral crusts along the orifice and the perturbations in the BSR depths) indicate that seepage in this location has been long-lived. While we do not know whether Pythia’s Oasis is the only seep of its kind, it is possible that similar seeps exist along the three strike-slip faults in the central CSZ. Therefore, strike-slip faults may play a consequential role in the regulation of overpressures throughout the forearc in the central CSZ and should be considered in future models of the CSZ.

## METHODS

### Multibeam sonar acquisition and processing

Multibeam sonar surveys were conducted above Pythia’s Oasis nine times over a 12-year period spanning 2014–2022 (fig. S10). All surveys were completed during University of Washington/National Science Foundation expeditions in support of the Ocean Observatories Initiative’s Regional Cabled Array; all acoustic surveys at Pythia’s Oasis took place between ROV dives or during adverse weather conditions. A dedicated seafloor mapping survey was conducted onboard the R/V *Thompson* in 2014 using the hull-mounted Kongsberg EM302 multibeam echosounder; all subsequent sonar surveys were focused on bubble plume detection through water column imaging using either a Kongsberg EM112 or EM302 system (table S1). Additional multibeam data were collected during a survey of the ACF onboard the R/V *Atlantis* during expedition AT 42-17; data from this survey are included in Fig. 1. Raw sonar files used in this study are available online through the Rolling Deck to Repository (R2R) program. Sound velocity profiles were derived from expendable bathythermograph casts conducted before each sonar survey, and typical vessel survey speeds ranged from 4 to 5 knots. Bathymetric data were processed using QPS Fledermaus DMagic (version 7.8.7), using a Combined Uncertainty and Bathymetry Estimator (CUBE) hypothesis method for a 15-m horizontal-resolution gridded surface. Water column data were processed using QPS Fledermaus Midwater (version 7.3.0b), enabling the isolation of bubble plume signals through the application of amplitude, beam, range, and ping filters.

### Submersible dives and sample preservation

Three ROV dives were conducted over a 3-year period using *ROPOS* (operated by the Canadian Scientific Submersible Facility) and *Jason* (operated by Woods Hole Oceanographic Institution; table S1). *ROPOS* dive R1858 (2015) was equipped with a Sea-Bird SBE 19plus CTD sensor, and ROV *Jason* dives J2-923 (2016) and J2-990 (2017) used a high-temperature thermistor to measure the temperature of the egressed fluid. High-definition video, collected using a forward-looking Insite Pacific Zeus-Plus HD camera during R1858, was used to provide a first-order estimate of fluid flow rates at Pythia’s Oasis through the application of basic video analysis software. A 7-s video clip (movie S2) was analyzed using Tracker 4.92 (an open-source video analysis and modeling tool) to track the advection of suspended sediment particles within two 15-frame sequences. Distances were calibrated on the basis of the diameter of the orifice (5 cm) measured using parallel lasers on the ROV spaced 10 cm apart.

Vent fluid samples were collected using a Major sampler (2016) and IGT sampler (2017) (*70*) onboard the ROV *Jason*; the latter preserves the sample at in situ pressures for subsampling onboard the ship, while the former allows gas to expand and escape the collection chamber during vehicle ascent and is therefore considered a less-pristine sample. Samples were transferred to 30-ml high-density polyethylene bottles, with a subset acidified (1% v/v) with trace metal-grade nitric acid. In 2016, two sediment push cores, with lengths of 12 and 20 cm, were collected 1 m away from the orifice. The cores were immediately refrigerated at 4°C and were preserved for shore-based processing. On shore, sediment pore waters were extracted at 4-cm intervals using Rhizon samplers and the fluids were split into acidified and nonacidified subsamples; pore waters designated for oxygen and hydrogen isotopic analysis were preserved in flame-sealed ampules, while the subsamples for sulfate analysis were preserved with zinc acetate to precipitate out dissolved sulfide (except for the samples collected using the IGT samplers in 2017).

### Chemical analyses

Cl concentrations were measured by colorimetric titration with AgNO_3_. Sulfate concentrations were determined using a Metrohm 882 Compact Ion Chromatograph Plus; standards were created by diluting International Association of Physical Sciences of the Ocean (IAPSO) standard seawater with 18.2-megohm milli-Q water. Chloride and sulfate were not determined in the IGT samples because of improper preservation. The concentrations of the major seawater cations (i.e., major elements; Na, Mg, Ca, and K) were measured within pore water and vent fluid samples, while minor elements (B and Li) were measured only within the IGT samples. Major and minor element concentrations were determined using a PerkinElmer 8300 Inductively Coupled Plasma Optical Emission Spectrometer. Major element samples were diluted 100× and calibrated using IAPSO standards diluted to 2.5 to 120% of seawater, while minor element samples were diluted 26× and calibrated using dilutions of Spex CertiPrep standards; all dilutions were made with nanopure water. Because the data presented are for discrete fluid samples from a focused vent site, we present errors as analytical accuracy. The accuracy is computed from the percent difference between reference standards: Ca < 1%, K < 3%, Mg <1.3%, Na <5%, SO_4_ < 1%, Cl < 0.3%, B < 2.5%, and Li <6%. Oxygen and hydrogen isotopes were measured using a Picarro L2130-i Cavity Ringdown Spectrometer and calibrated to Vienna standard mean ocean water and in-house standards; pore water uncertainties were 0.07‰ δ^18^O and 0.20‰ δD.

In addition, all measurements made on samples collected from Major samplers, including major and minor elements, were corrected for mixing with seawater entrained near the vent orifice. Mixing calculations assume an end-member with zero sulfate, a likely scenario given the complete consumption of sulfate within pore water below the SMTZ (*71*), which is positioned at ~15-cm depth within the sediments at Pythia’s Oasis.

### MCS data acquisition

MCS data were collected onboard the R/V *Revelle* in October of 2017 during a National Science Foundation Chief Scientist Training Cruise (RR1718). Seismic data were collected using the Scripps Institution of Oceanography portable high-resolution MCS system, consisting of an 800-m-long (600 m active) 48-channel Geometrics GeoEel streamer (12.5-m spacing per channel) and a two–Sercel GI 210 Air Gun array, operating in a 45/105 “True GI Mode” configuration. Shots were spaced at 25-m intervals, with a sampling rate of 0.5 ms and a record length of 8.0 s. Data were collected at an average vessel speed of 4.5 kts, with a total of 45 km of seismic data collected above Pythia’s Oasis (Fig. 1). Seismic lines used in this study are available through the Marine Geoscience Data System archive.

### MCS data processing

Seismic Unix was used to apply a 5-octave band-pass filter to remove low- and high-frequency noise. Channels 1, 17, and 33 were found to be consistently noisy across all seismic lines and were muted for subsequent analyses. A maximum fold of 12 for the survey data resulted from 12.5-m receiver spacing, 25-m shot spacing, and 45 unmuted hydrophones. This fold did not result in substantial normal move out, and semblance analysis was limited to within 0.5-s TWT of the seafloor. To minimize introduced error from a velocity model derived from limited semblance information, all seismic data were stacked and migrated using a constant velocity of 1500 m s^−1^.

BSRs were picked by loading the migrated seismic lines into Kingdom 8.8 and by applying the following criteria: (i) BSRs are negative polarity with respect to the seafloor, which is consistent with a decrease in seismic p-wave velocity coincident with the transition from solid gas hydrates to free gas below; (ii) BSRs cross-cut strata, minimizing the likelihood of mis-identifying a lithologic boundary; and (iii) the BSR is picked only where the reflector can be clearly distinguished from gas-rich regions beneath the seep. BSRs were picked using the negative-polarity horizon with an estimated uncertainty of 5.0 ms or ~7.5 m. Seafloor and BSR picks were converted from TWT to depth using the constant velocity model.

### Geothermal gradient and heat flow calculations

Estimation of the geothermal gradient from BSR observations requires measurement of the TWT to the seafloor and the BSR, as well as data on the seafloor temperature, the phase boundary between free and gas hydrate for the local gas and pore water composition, the seismic velocity of the sediments hosting the BSR, and subseafloor pressure regime, which is likely between hydrostatic and lithostatic. Conversion of the geothermal gradient to heat flow is sensitive to the thermal conductivity of sediments overlying the BSR (*41*–*42*). Here, we present heat flow values based on a range of thermal conductivity values to account for the dominant source of uncertainty in this calculation.

Surface heat flow was calculated using a one-dimensional solution to Fourier’s law

$$q=-k\frac{T_{\mathrm{bsr}}-T_{\mathrm{sf}}}{z_{\mathrm{bsr}}-z_{\mathrm{sf}}}$$

where *q* is the heat flow, *k* is the thermal conductivity of the sediments, *T*_bsr_ and *T*_sf_ are the temperatures at the BSR and seafloor, respectively, and *z*_bsr_ and *z*_sf_ are the depth of the BSR and seafloor (meters below sea level), respectively. To account for the sensitivity of heat flow calculations to thermal conductivity, heat flow was calculated using two values for thermal conductivity, resulting in a minimum heat flow value (using 0.9 W m^−1^ °C^−1^ and hydrostatic pressure) and a maximum heat flow value (using 1.15 W m^−1^ °C^−1^ and lithostatic pressure); these thermal conductivities were from measurements made at ODP Site 1252 (*72*), located 11 km north of Pythia’s Oasis. Seafloor and BSR depths were determined using the seismic time picks and a constant-velocity (1500 m s^−1^) time-to-depth conversion; a sensitivity analysis conducted to constrain the dependence of heat flow values on the seismic velocity used for the time-to-depth conversion indicates a 1.8% difference between heat flow values calculated for 1500 and 1800 m s^−1^, in line with previous estimates of heat flow uncertainties resulting from various seismic velocities (*42*). Seafloor temperatures at Pythia’s Oasis were estimated using bottom water temperature outputs from a regional ocean circulation model over a 4-year (2010–2014) simulation (*73*); 3.3° ± 0.1°C represent the mean and SD over the modeled period. This model was verified using bottom water temperature measurements from ocean bottom seismometers deployed at several locations along the central Oregon margin from 2012 to 2013 for the Cascadia Amphibious Array.

In situ BSR temperatures are dependent on in situ pore pressures, hydrate composition, and pore water chlorinity (*74*). Previous estimates of pore pressures at the BSR in Cascadia have spanned from fully hydrostatic (*29*, *51*, *75*–*77*), partially lithostatic (*78*), to fully lithostatic (*79*). While purely hydrostatic conditions would be insufficient to drive the observed fluid flow at Pythia’s Oasis, fully lithostatic conditions are rarely achieved in shallow, hydrologically active seafloor sediments. To account for the uncertainty in in situ pore pressures, we calculate in situ temperatures (and therefore geothermal gradients and heat flow values) in two scenarios: one being fully hydrostatic (where the modified pore pressure ratio, λ*, is zero) and the other being fully lithostatic (λ* equal to 1) using the following equation

$$P_{\mathrm{bsr}}={g\rho }_{\mathrm{sw}}z_{sf}+\lambda^{*}g\rho_{s}z_{\mathrm{bsr}}+(1-\lambda^{*})g\rho_{\mathrm{sw}}z_{\mathrm{bsr}}$$

where *z*_sf_ represents the depth of the seafloor below sea level and *z*_bsr_ represents the depth of the BSR below the seafloor. Seawater density (ρ_sw_) was measured via CTD during an ROV ascent above Pythia’s Oasis in 2015, where the mean and SD of the seawater density was 1028.5 ± 2.7 kg m^−3^. This density profile was extended beyond the lowest sampled depth (1043 mbsl) using a linear fit to the lower half of the water column profile (*R*^2^ = 0.9998), allowing for the calculation of seafloor pressures at deeper locations. Sediment density (ρ_s_) was calculated using bulk density measurements from ODP Site 1252 (*72*). A 1-m resolution model of bulk sediment density as a function of depth was created via linear interpolation of ODP measurements made at 0.05- to 6.9-m intervals (*72*). The overlying bulk sediment mass at the BSR was calculated by numerically integrating this model at 1-m intervals.

The temperature at the BSR was determined using the calculated in situ pore pressures and a theoretical gas hydrate stability model (*80*). A structure-I hydrate (i.e., pure methane) was assumed, which is consistent with samples recovered during ODP drilling at the summit of Southern Hydrate Ridge (located 10 km northwest of Pythia’s Oasis) that indicates methane as the dominant hydrocarbon within the pore water (*81*). A pore water salinity of 35 PSU was used to initiate the model, in agreement with observations of pore water chlorinity at the BSR equal to that of seawater (550 mM Cl^−^) (*50*).

### Estimating the duration of focused flow from BSR perturbation

For a first-order estimate of the minimum length of time that this seep has been active, we calculated the time required for the lateral diffusion of heat from a vertical conduit to perturb temperatures at a given distance from the seep, using the closest-detectable BSR as a reference point relative to background values. To calculate the lateral diffusion of heat from a point source, we used the following analytical solution to the one-dimensional heat transport equation

$$\Delta T(t,L)=\Delta T_{0}erfc\left(\frac{L}{2\sqrt{\kappa t}}\right)\;\text{where}\;\kappa =\frac{\lambda }{\rho c}$$

where Δ*T*_0_ is the temperature of the fluid at time (*t*) zero, *L* is the horizontal distance between the temperature source and the point being modeled, λ is thermal conductivity, ρ is fluid density, and *c* is the fluid heat capacity.

For the purposes of this calculation, the following assumptions were made: a fluid source of 20°C, a thermal diffusivity of 1.5 × 10^−7^ m^2^ s^−1^ (which was calculated assuming a thermal conductivity of 1 W m^−1^ K^−1^, a fluid density of 1000 kg m^−3^, and fluid heat capacity of 4000 J kg^−1^ K^−1^), and an instantaneous step change in temperature at the boundary. Although it is several degrees warmer than the 11.8° to 12.6°C measured at the orifice, the 20°C source temperature was chosen given likely mixing with bottom water near the seafloor and because it would result in a conservative estimate of the length of time that the seep has been active, given that lower source temperatures would require greater time scales to perturb the BSR at distance.

The depth and temperature of the BSR in background locations was compared to the temperature at comparable depths near the seep, using the closest-detectable BSR to the seep that is roughly 150 m away laterally. The geothermal gradient at that location was used to determine that temperatures were elevated ~6.5°C above temperatures at the same depth below seafloor as the BSR in background locations, representing the in situ change in temperature over time due to the onset of vertical flow.

The amount of time required for the in situ temperature to increase by 6.5°C was determined by interpolating between diffusion curves (fig. S7), centered on this temperature and the distance from the source (150 m). The closest match was ~1500 years, which is a conservative estimate given that (i) it is difficult to imagine a perfect conduit, and (ii) it is unlikely that flow is continuous, and intermittent flow would require greater time scales to perturb the BSR to the degree detected at this location.

## Supplementary Materials

### This PDF file includes:

Figs. S1 to S10 Tables S1 and S2 References
