# Supplementary material for: Fluid sources and overpressures within the central Cascadia Subduction Zone revealed by a warm, high-flux seafloor seep
Source: Sci Adv. 2023 Jan 25;9(4):eadd6688. doi: 10.1126/sciadv.add6688 (PMC9876559; doi:10.1126/sciadv.add6688)
Supplement: Supplementary file 1 — Figs. S1 to S10 Tables S1 and S2 References [file sciadv.add6688_sm.pdf]

Science Advances
AAAS

Supplementary Materials for

Fluid sources and overpressures within the central Cascadia Subduction Zone revealed by a warm, high-flux seafloor seep

Brendan T. Philip *et al.*

Corresponding author: Brendan T. Philip, brendan.t.philip@gmail.com

Sci. Adv. **9**, eadd6688 (2023)
DOI: 10.1126/sciadv.add6688

The PDF file includes:

Figs. S1 to S10
Tables S1 and S2
Legends for movies S1 and S2
References

Other Supplementary Material for this manuscript includes the following:

Movies S1 and S2

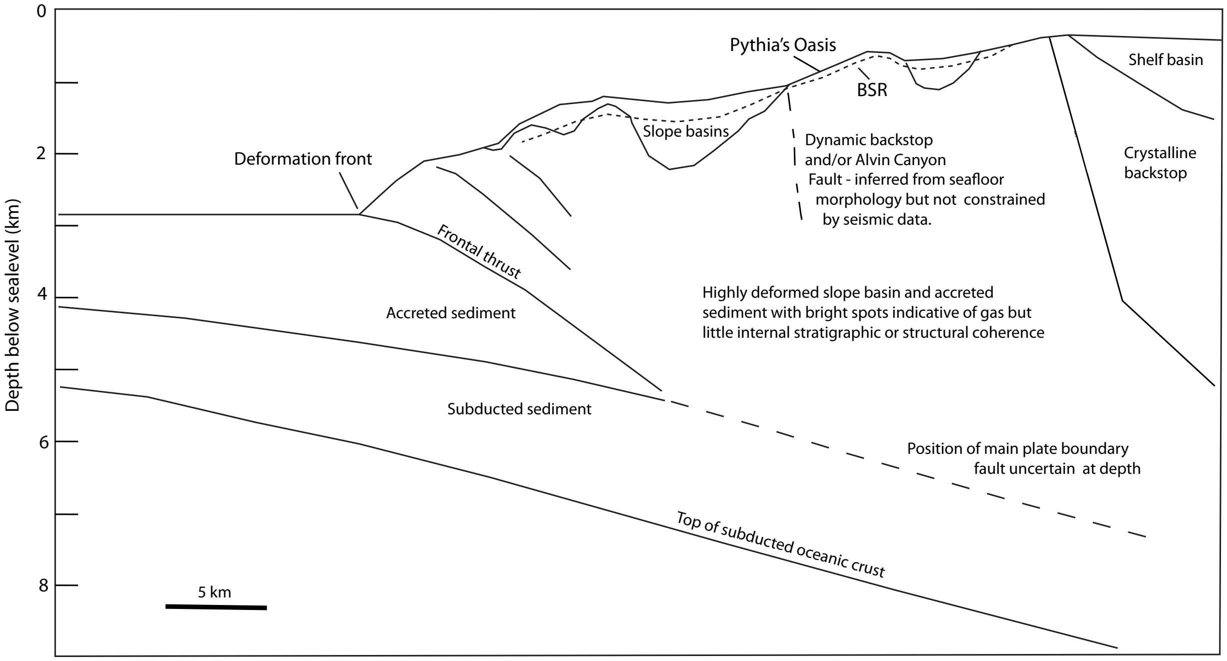

Figure S1. Schematic illustration of the geologic and tectonic setting of *Pythia's Oasis* based on seafloor morphology, regional seismic reflection and large aperture (refraction) data (*33*). Amphibious seismic imaging indicates that the top of the subducted oceanic crust is at a depth of at least 7 km beneath *Pythia's Oasis*. All of the seismic reflection lines that cross the deformation front in this region show a landward-dipping frontal thrust that soles into a stratigraphically defined decollement, with approximately ½ the incoming sediment accreted to the margin in a series of folds and thrust and ½ of the incoming sediment subducted beneath the margin. However, the decollement is not imaged more than ~10 km east of the deformation front, likely because of strong scattering with the highly deformed accreted sediment wedge. Whether the subducted sediment is underplated to the base of the accretionary wedge or subducted more deeply beneath the margin remains poorly known. Two major structural boundaries based on seafloor morphology but inferred to extend through the accretionary wedge have been mapped in the vicinity of Pythia's Oasis. The Alvin Canyon Fault is manifested by a pronounced left lateral offset in the deformation front and is thought to be steeply dipping (*64*). The dynamic backstop is defined by a margin-wide change in the slope of the accretionary wedge and is interpreted to be a splay fault originating on or near the plate boundary and separating the Pleistocene from the older accretionary wedge. Either (or both) of these structures, if present, is a potential conduit for fluids from compressed and dehydrated subducted sediment, but we did not find any conclusive evidence of either structure in the regional seismic reflection data.

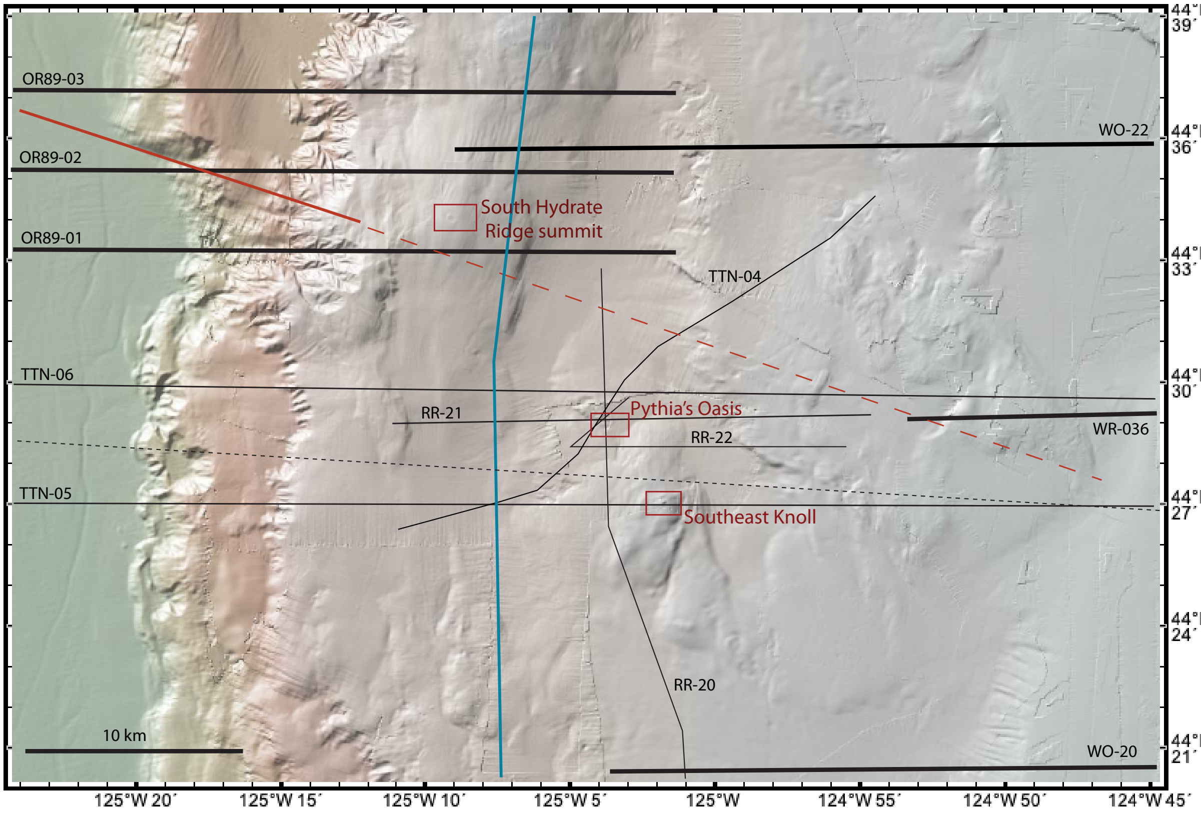

Figure S2. Map showing regional seafloor morphology and the spatial relationship between *Pythia's Oasis*, existing seismic reflection data and inferred major tectonic structures. Red line is the Alvin Canyon Fault (*64, 67*). Teal line is the dynamic backstop (*83*). Bold black lines are crustal scale data. Fine black lines are high resolution data. WR-36 is from a 1975 WesternGeco regional survey. WO-20 and 22 are from a 1980 WesternGeco survey. Data are available from the USGS NAMSS web site. OR89-01, 02 and 03 are from the 1989 ODP Leg 146 site survey (*29*). TTN-04, 05 and 06 are from cruise TTN112, conducted as a site survey for ODP Leg 204. RR-20, 21 and 22 are from cruise RR1718. All seismic data are available from the USGS NAMSS (WesternGeco) or Academic Seismic Portal (OR89, TTN and RR) and were reviewed for possible evidence of deeper structure in the vicinity of *Pythia's Oasis*. Note: additional high-resolution data acquired over Hydrate Ridge and north of TTN-06 during cruises TTN112 and EW02-08 exist but are not included on this map because they were acquired to image the structure beneath Hydrate Ridge and do not extend south of TTN-06.

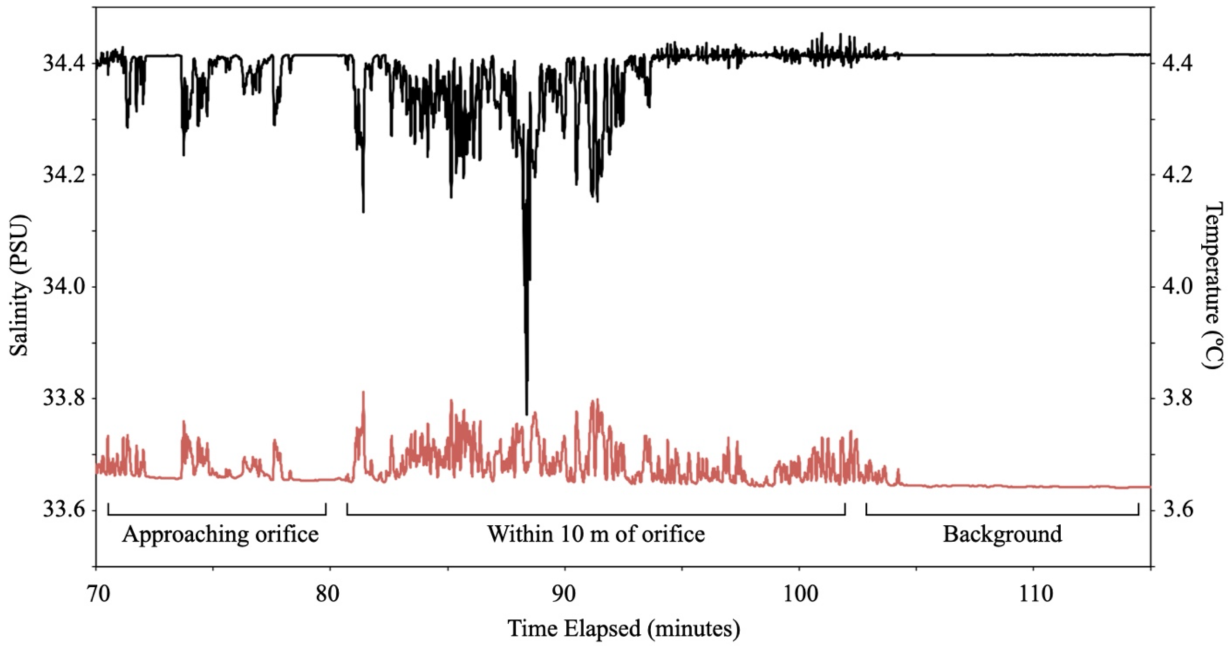

Fig. S3. Measurements of seawater temperature (red line) and salinity (black line) made using the SBE 19plus CTD onboard the ROV ROPOS during dive R1858 in 2015. The timeseries data shown here were collected while the ROV was in the vicinity of the orifice at *Pythia's Oasis*; the position of the ROV with respect to the orifice is labeled.

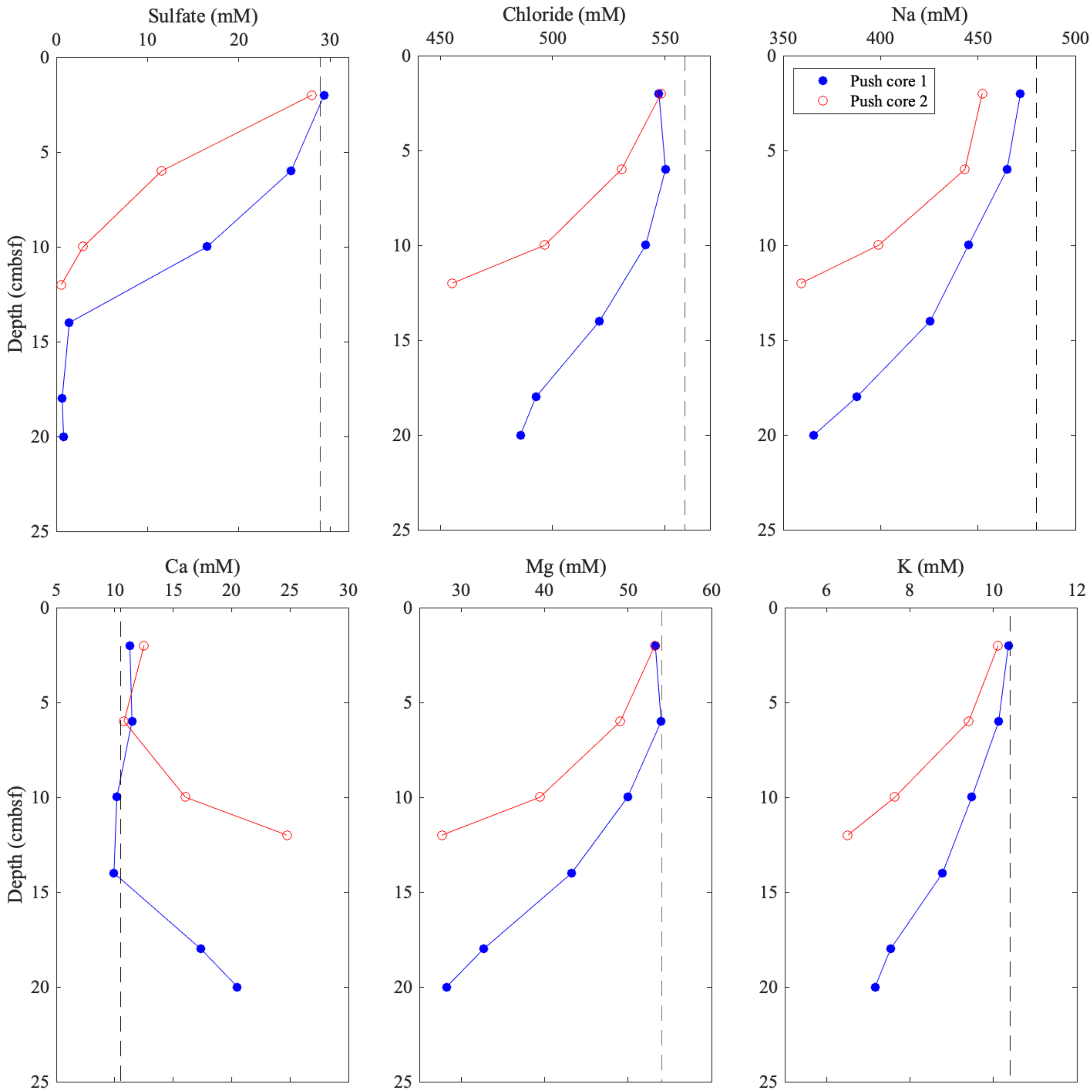

Fig. S4. Pore water profiles derived from push cores collected ~1 m from the site of focused flow at *Pythia's Oasis.* Seawater values are indicated by a vertical, dashed gray line.

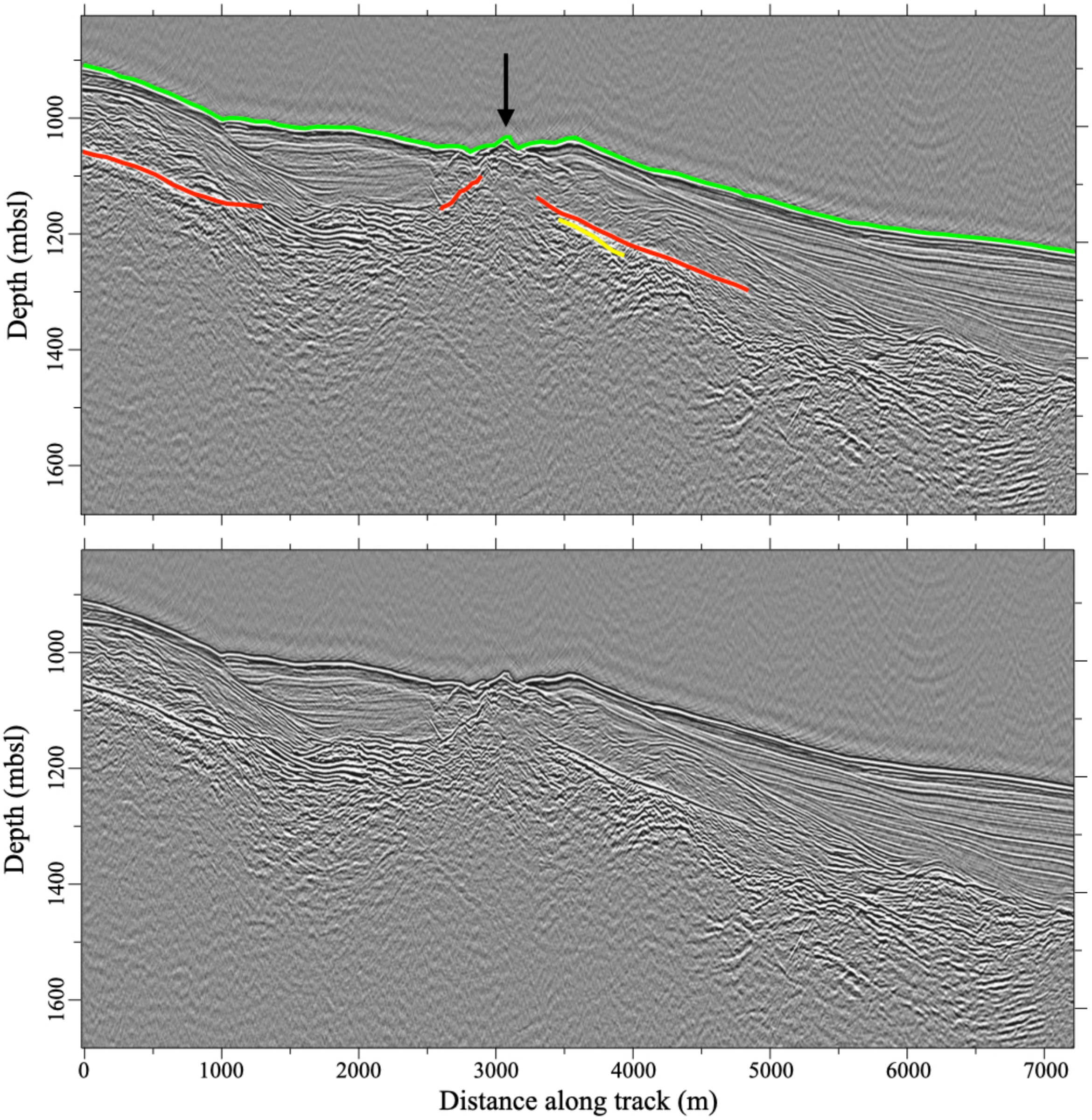

Fig. S5. (Top) Seismic line 20 - looking west - with the seafloor (green), bottom simulating reflectors (red) and a possible secondary BSR (yellow) highlighted. Vertical arrow indicates location of *Pythia's Oasis*. (Bottom) Seismic line 20 without interpretations.

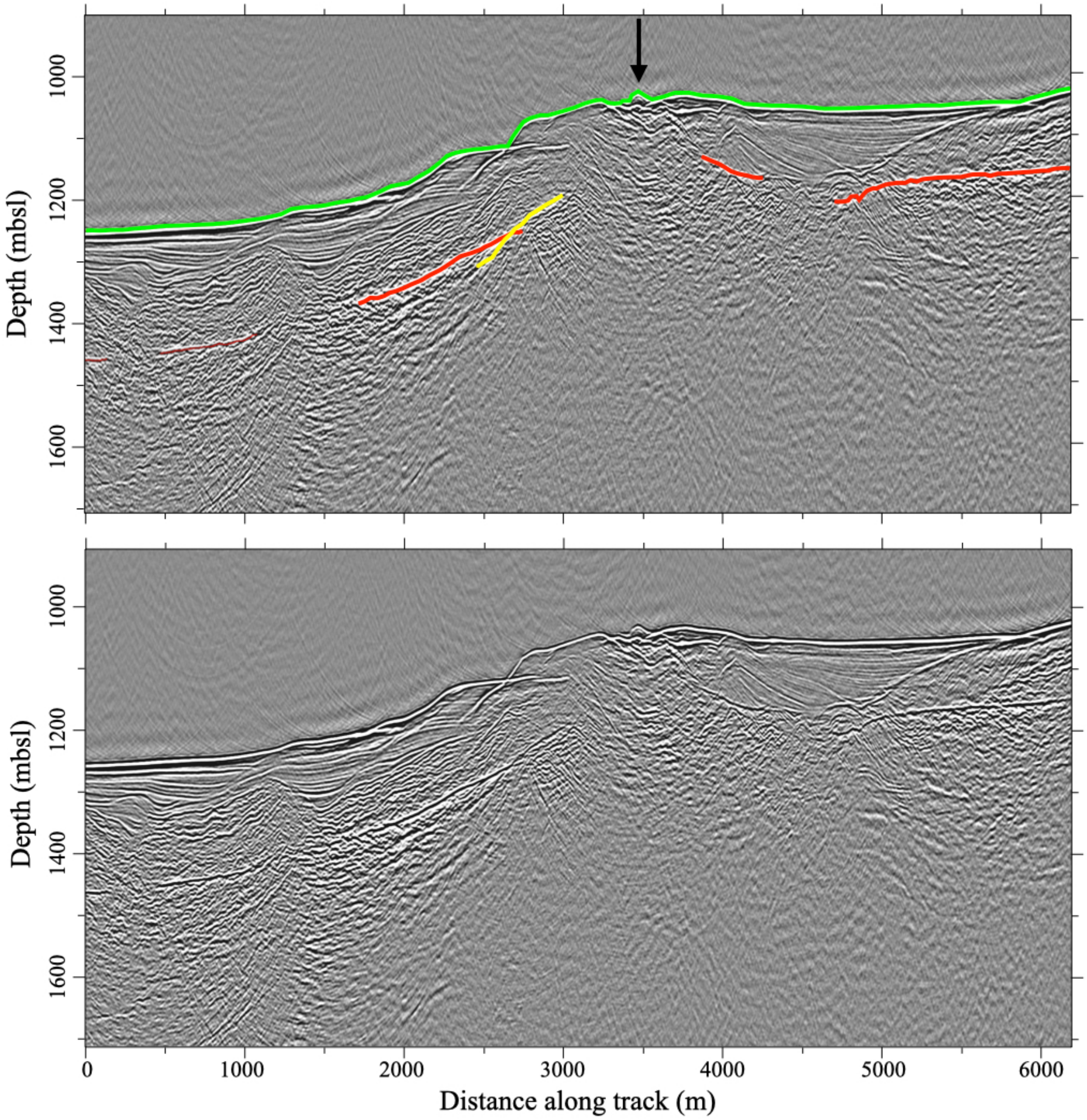

Fig. S6. (Top) Seismic line 21 - looking north - with the seafloor (green), bottom simulating reflectors (red) and a possible secondary BSR (yellow) highlighted. Vertical arrow indicates location of *Pythia's Oasis*. (Bottom) Seismic line 21 without interpretations.

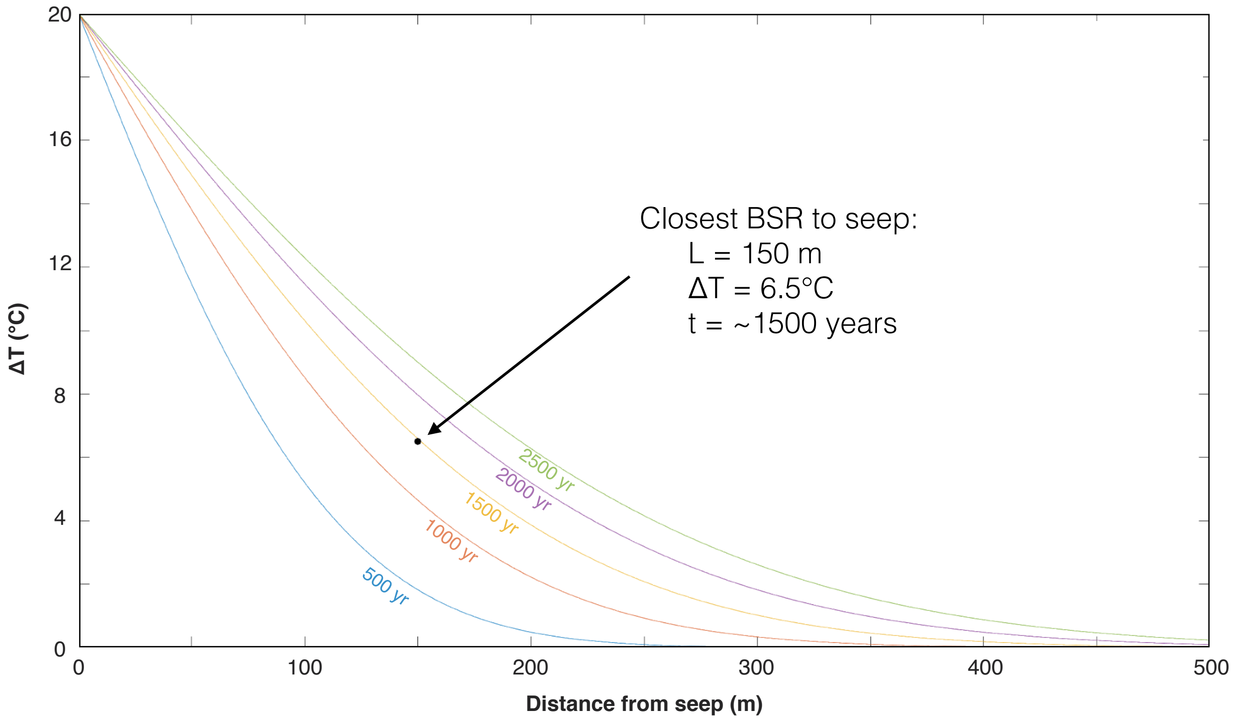

Figure S7. Results of the 1-dimensional heat transport model used to provide a first-order estimate of the duration of focused flow at *Pythia's Oasis*. See Methods for the assumptions used in this calculation. The curves shown here represent the *in situ* temperature as a function of distance from the seep that would result from sustained vertical flow of 20 °C fluid for 500-2500 years.

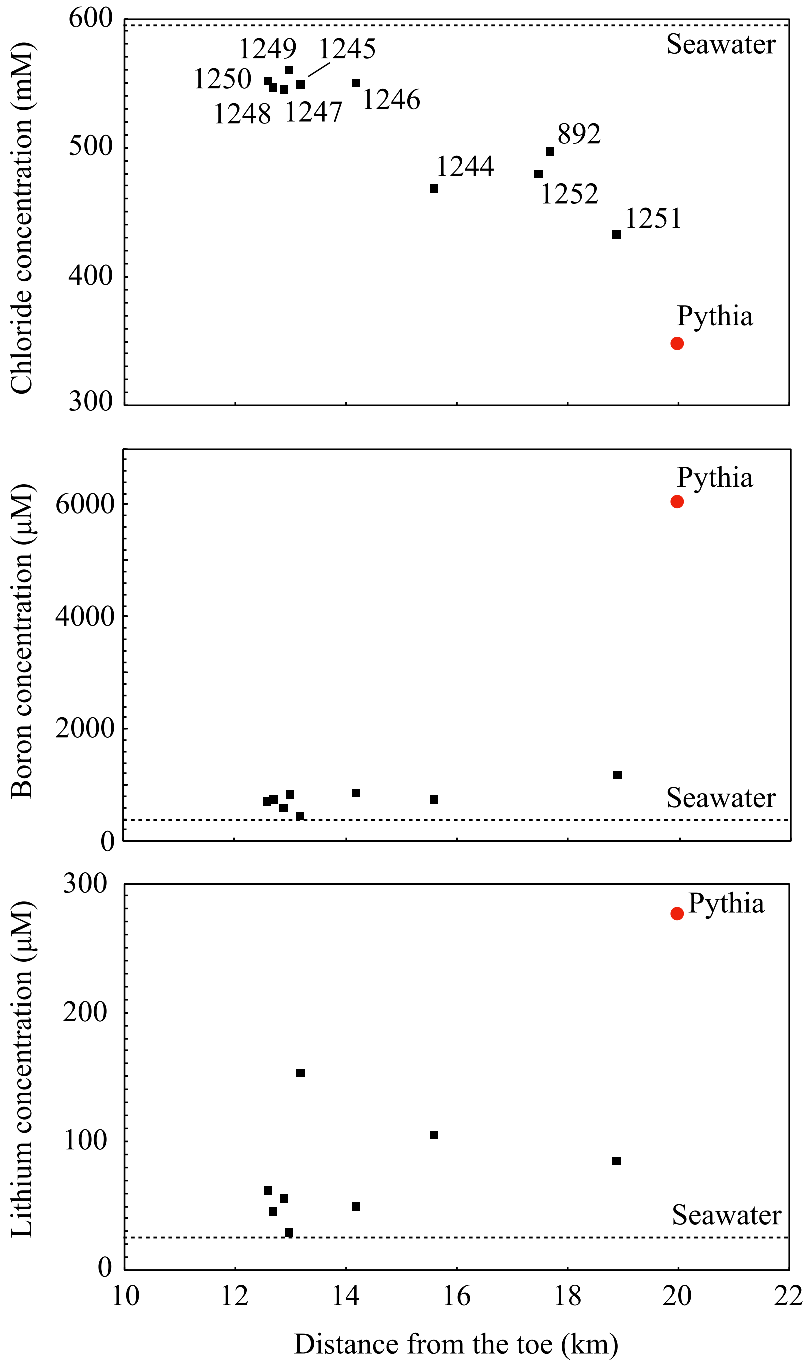

Fig. S8. Pore water composition as a function of distance landward from the toe of the accretionary prism in Central Oregon. Displayed are pore water concentrations from the deepest sections (86–467 mbsf) of ODP holes drilled during Leg 146 (*32*) and Leg 204 (*33*). Also displayed are the mean values from samples collected at *Pythia's Oasis*: the chloride concentration is from samples collected using Major samplers in 2016, and the lithium and boron data are from the IGT samples collected in 2017. The Li and B concentrations shown for *Pythia's Oasis* are uncorrected for mixing with seawater due to a lack of sulfate concentration data. Approximate seawater values are indicated by dashed horizontal lines.

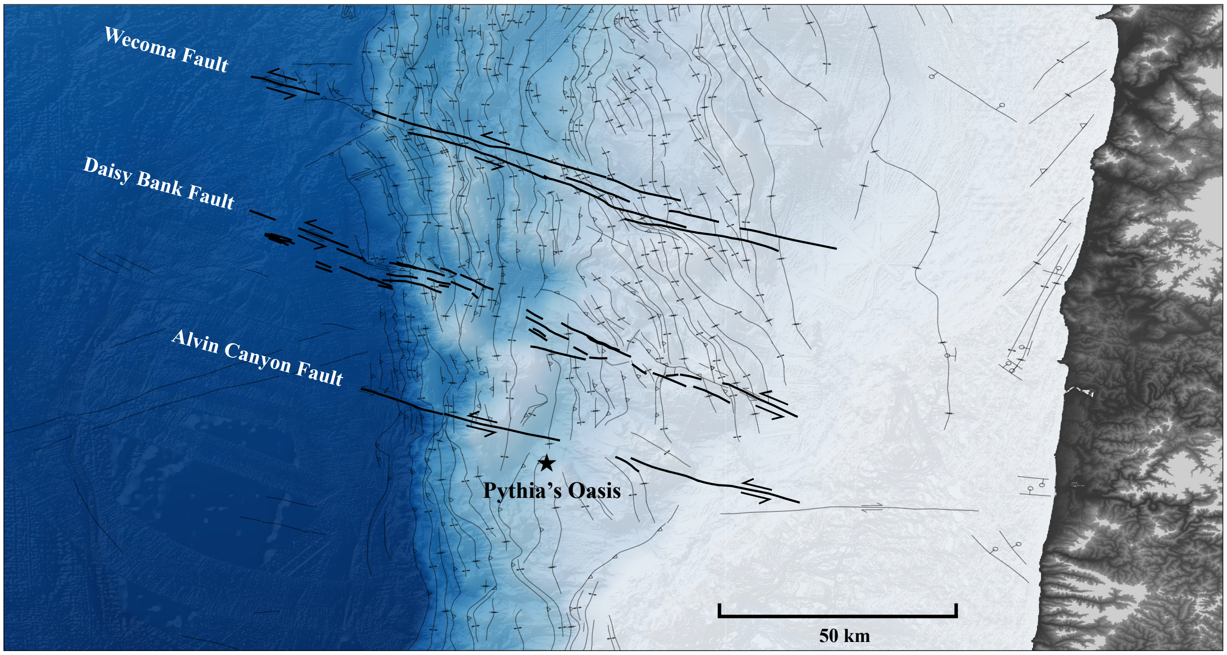

Fig. S9. Location of *Pythia's Oasis* relative to the three strike-slip faults that crosscut the margin in central Oregon (*64*). Bathymetry displayed is that of GMRT Version 3.7 (October 2019) (*82*) and fault traces are drawn from previous geophysical surveys (*67*).

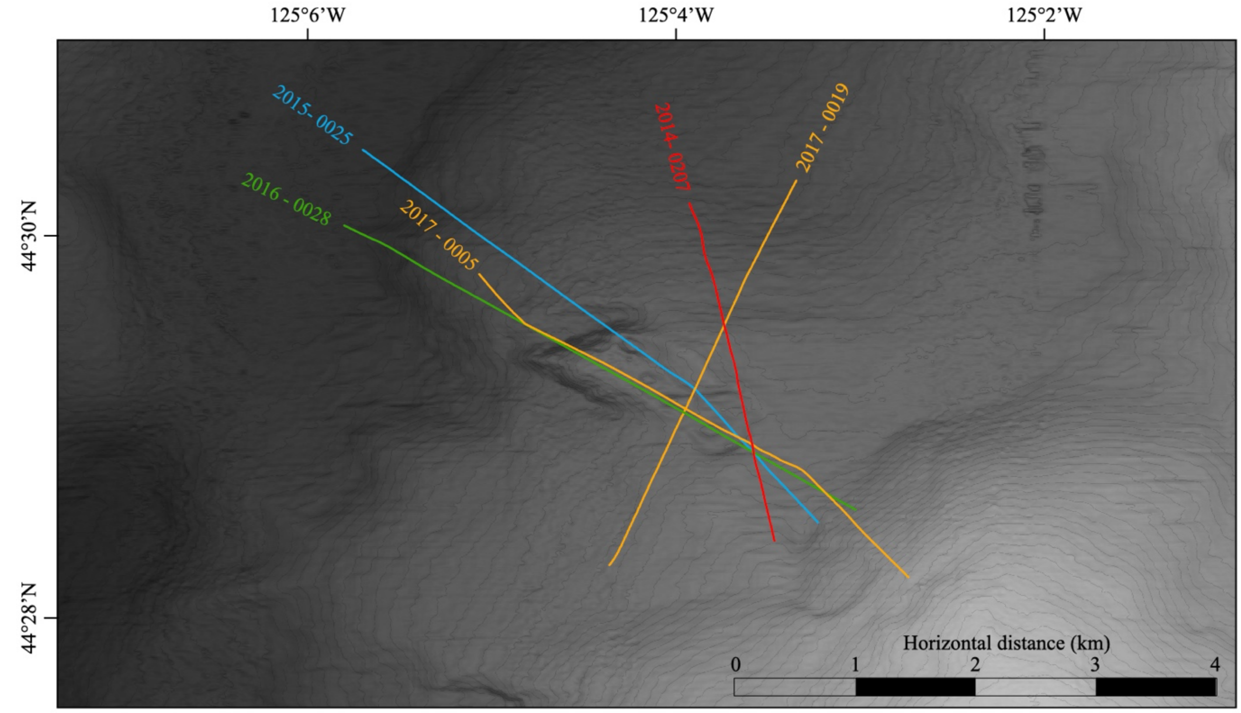

Fig. S10. Vessel track lines during multibeam sonar surveys in 2014 (red), 2015 (blue), 2016 (green), and 2017 (orange); for 2018 survey line, see Fig. 1 in the main text. The sonar file name follows the year in each label. For specifics on survey vessel and data availability, see Table S1.

Table S1. Summary of Surveys and ROV Dives completed at *Pythia's Oasis*, 2014-2018

Year	Vessel	Expedition	Survey Type	Survey Date (UTC) MM/DD/YYYY	File*
2014	R/V *Thompson*	TN313	EM302	09/02/2014	0090
			EM302	09/06/2014	0207
2015	R/V *Thompson*	TN326	EM302	07/15/2015	0025
			ROPOS-R1858	07/24/2015	-
2016	R/V *Sikuliaq*	SKQ201610S	EM302	07/17/2016	0082
			Jason-J2-923	07/20/2016	-
2017	R/V *Sikuliaq*	SKQ201705S	EM302	04/28/2017	0019
	R/V *Revelle*	RR1713	Jason-J2-990	08/08/2017	-
		RR1714	EM122	08/11/2017	0005
		RR1718	MCS	10/1/2017	20, 21
2018	R/V *Revelle*	RR1809	EM122	06/25/2018	0015

*Raw sonar files are available via the R2R repository (https://www.rvdata.us) and are organized by expedition ID. MCS files are available through the MGDS database (http://www.marine-geo.org) under Expedition RR1718.

Table S2. Major and IGT sample composition.

	**Major**	**IGT-1**	**IGT-2**	**Seawater**†
**Salinity (psu)**	21.4	-	-	35
**Chloride (mM)**	347	-	-	559
**Sulfate (mM)**	5.8*	-	-	28.9
**Ca (mM)**	53.4	50.5	50.3	10.5
**K (mM)**	2.1	2.2	2.2	10.4
**Mg (mM)**	5.1	7.3	7.3	54
**Na (mM)**	245	209	211	480
**B (µM)**	-	5952.3	6100	420
**Li (µM)**	-	273.6	277	26
**δD (‰)**	-10.4	-8.8	-	0
**δ18O (‰)**	-0.1	0.5	-	0

*The Major sulphate concentration represents the pre-mixing-corrected value.
†Seawater concentrations represent average values; bottom water was not collected at *Pythia's Oasis.*

Movie S1.
This video was recorded during the first dive to the seafloor at *Pythia's Oasis* during *ROPOS* dive R1858 on 24 July 2015.

Movie S2.
This seven-second video was used to estimate water flow rates from the orifice at *Pythia's Oasis*. The footage was recorded during *ROPOS* dive R1858 on 24 July 2015.

REFERENCES AND NOTES

1. J. C. Moore, D. Saffer, Updip limit of the seismogenic zone beneath the accretionary prism of southwest Japan: An effect of diagenetic to low-grade metamorphic processes and increasing effective stress. *Geology* **29**, 183–186 (2001).

2. C. R. Ranero, I. Grevemeyer, H. Sahling, U. Barckhausen, C. Hensen, K. Wallmann, W. Weinrebe, P. Vannucchi, R. von Huene, K. McIntosh, Hydrogeological system of erosional convergent margins and its influence on tectonics and interplate seismogenesis. *Geochem. Geophys. Geosyst.* **9**, Q03S04 (2008).

3. D. M. Saffer, H. J. Tobin, Hydrogeology and Mechanics of Subduction Zone Forearcs: Fluid Flow and Pore Pressure. *Annu. Rev. Earth Planet. Sci.* **39**, 157–186 (2011).

4. T. Sun, S. Ellis, D. Saffer, Coupled evolution of deformation, pore fluid pressure, and fluid flow in shallow subduction forearcs. *J. Geophys. Res. Solid Earth* **125**, e2019JB019101 (2020).

5. S. Ellis, A. Fagereng, D. Barker, S. Henrys, D. Saffer, L. Wallace, C. Williams, R. Harris, Fluid budgets along the northern Hikurangi subduction margin, New Zealand: The effect of a subducting seamount on fluid pressure. *Geophys. J. Int.* **202**, 277–297 (2015).

6. B. A. Bekins, S. J. Dreiss, A simplified analysis of parameters controlling dewatering in accretionary prisms. *Earth Planet. Sci. Lett.* **109**, 275–287 (1992).

7. W. G. Ernst, Thermobarometric and fluid expulsion history of subduction zones. *J. Geophys. Res.* **95**, 9047–9053 (1990).

8. M. Kastner, E. A. Solomon, R. N. Harris, M. E. Torres, Fluid origins, thermal regimes, and fluid and solute fluxes in the forearc of subduction zones, in *Earth and Life Processes Discovered Beneath the Subseafloor*, R. Stein, D. K. Blackman, F. Inagaki, H. C. Larsen, Eds. (Elsevier, 2014), vol. 7, pp. 671–733.

9. G. A. Spinelli, D. M. Saffer, Along-strike variations in underthrust sediment dewatering on the Nicoya margin, Costa Rica related to the updip limit of seismicity. *Geophys. Res. Lett.* **31**, L04613 (2004).

10. D. M. Saffer, M. B. Underwood, A. W. McKiernan, Evaluation of factors controlling smectite transformation and fluid production in subduction zones: Application to the Nankai Trough. *Island Arc* **17**, 208–230 (2008).

11. D. M. Saffer, The permeability of active subduction plate boundary faults. *Geofluids* **15**, 193–215 (2015).

12. M. K. Hubbert, W. W. Rubey, Role of fluid pressure in mechanics of overthrust faulting. *Bull. Geol. Soc. Am.* **70**, 115–166 (1959).

13. P. Audet, M. G. Bostock, N. I. Christensen, S. M. Peacock, Seismic evidence for overpressured subducted oceanic crust and megathrust fault sealing. *Nature*, **457**, 76–78 (2009).

14. R. M. Lauer, D. M. Saffer, The impact of splay faults on fluid flow, solute transport, and pore pressure distribution in subduction zones: A case study offshore the Nicoya Peninsula, Costa Rica. *Geochem. Geophys. Geosyst.* **16**, 1089–1104 (2015).

15. N. L. B. Bangs, K. D. McIntosh, E. A. Silver, J. W. Kluesner, C. R. Ranero, Fluid accumulation along the Costa Rica subduction thrust and development of the seismogenic zone. *J. Geophys. Res. Solid Earth* **120**, 67–86 (2015).

16. B. A. Bekins, A. M. McCaffrey, S. J. Dreiss, Episodic and constant flow models for the origin of low-chloride waters in a modern accretionary complex. *Water Resour. Res.* **31**, 3205–3215 (1995).

17. L.-H. Chan, M. Kastner, Lithium isotopic compositions of pore fluids and sediments in the Costa Rica subduction zone: Implications for fluid processes and sediment contribution to the arc volcanoes. *Earth Planet. Sci. Lett.* **183**, 275–290 (2000).

18. E. Silver, M. Kastner, A. Fisher, J. Morris, K. McIntosh, D. Saffer, Fluid flow paths in the Middle America Trench and Costa Rica margin. *Geology*, **28**, 679–682 (2000).

19. A. J. Spivack, M. Kastner, B. Ransom, Elemental and isotopic chloride geochemistry and fluid flow in the Nankai Trough. *Geophys. Res. Lett.* **29**, 6-1–6-4 (2002).

20. E. A. Solomon, M. Kastner, G. Wheat, H. W. Jannasch, G. Robertson, E. E. Davis, J. D. Morris, Long-term hydrogeochemical records in the oceanic basement and forearc prism at the Costa Rica subduction zone. *Earth Planet. Sci. Lett.,* **282**, 240–251 (2009).

21. C. Hensen, K. Wallmann, M. Schmidt, C. R. Ranero, E. Suess, Fluid expulsion related to mud extrusion off Costa Rica—A window to the subducting slab. *Geology* **32**, 201–204 (2004).

22. R. M. Lauer, D. M. Saffer, Fluid budgets of subduction zone forearcs: The contribution of splay faults. *Geophys. Res. Lett.* **39**, L13604 (2012).

23. B. D. Cozzens, G. A. Spinelli, A wider seismogenic zone at Cascadia due to fluid circulation in subducting oceanic crust. *Geology* **40**, 899–902 (2012).

24. Y. Yokota, T. Ishikawa, S. Watanabe, T. Tashiro, A. Asada, Seafloor geodetic constraints on interplate coupling of the Nankai Trough megathrust zone. *Nature* **534**, 374–377 (2016).

25. L. M. Wallace, J. Beavan, R. McCaffrey, D. Darby, Subduction zone coupling and tectonic block rotations in the North Island, New Zealand. *J. Geophys. Res.* **109**, B12406 (2004).

26. G. M. Schmalzle, R. McCaffery, K. C. Creager, Central Cascadia subduction zone creep. *Geochem. Geophys. Geosyst.* **15**, 1515–1532 (2014).

27. K. Wang, A. M. Tréhu, Invited review paper: Some outstanding issues in the study of great megathrust earthquakes—The Cascadia example. *J. Geodyn.* **98**, 1–18 (2016).

28. S. Han, N. L. Bangs, S. M. Carbotte, D. M. Saffer, J. C. Gibson, Links between sediment consolidation and Cascadia megathrust slip behaviour. *Nat. Geosci.* **10**, 954–959 (2017).

29. G. Zwart, J. C. Moore, G. R. Cochrane, Variations in temperature gradients identify active faults in the Oregon accretionary prism. *Earth Planet. Sci. Lett.* **139**, 485–495 (1996).

30. N. L. B. Bangs, M. J. Hornbach, C. Brendt, The mechanics of intermittent methane venting at South Hydrate Ridge inferred from 4D seismic surveying. *Earth Planet. Sci. Lett.* **310**, 105–112 (2011).

31. G. J. Crutchley, C. Berndt, S. Geiger, D. Klaeschen, C. Papenberg, I. Klaucke, M. J. Hornbach, N. L. B. Bangs, C. Maier, Drivers of focused fluid flow and methane seepage at south Hydrate Ridge, offshore Oregon, USA. *Geology*, **41**, p. 551–554 (2013).

32. G. K. Westbrook, B. Carson, R. J. Musgrave, *Proceedings of the Ocean Drilling Program, Initial Reports, 146 (Pt. 1)* (Ocean Drilling Program, 1994).

33. A. M. Tréhu, M. E. Torres, G. Bohrmann, F. S. Colwell, Leg 204 synthesis: Gas hydrate distribution and dynamics in the central Cascadia accretionary complex, in *Proceedings of the Ocean Drilling Program, Scientific Results, 204*, A. M. Tréhu, G. Bohrmann, M. E. Torres, F. S. Colwell, Eds. (Ocean Drilling Program, 2006), pp. 1–41.

34. B. Carson, E. Suess, J. C. Strasser, Fluid flow and mass flux determinations at vent sites on the cascadia margin accretionary prism. *J. Geophys. Res.*, **95**, 8891–8897 (1990).

35. J. C. Moore, D. Orange, L. V. D. Klum, Interrelationship of fluid venting and structural evolution: *Alvin* observations from the frontal accretionary prism, Oregon. *J. Geophys. Res.* **95**, 8795–8808 (1990).

36. M. E. Torres, R. W. Embley, S. G. Merle, A. M. Tréhu, R. W. Collier, E. Suess, K. U. Heeschen, Methane sources feeding cold seeps on the shelf and upper continental slope off central Oregon, USA. *Geochem. Geophys. Geosyst.* **10**, Q11003 (2009).

37. T. Baumberger, R. W. Embley, S. G. Merle, M. D. Lilley, N. A. Raineault, J. E. Lupton, Mantle-derived helium and multiple methane sources in gas bubbles of cold seeps along the cascadia continental margin. *Geochem. Geophys. Geosyst.*, **19**, 4476–4486 (2018).

38. E. A. Schauble, G. R. Rossman, H. P. Taylor Jr., Theoretical estimates of equilibrium chlorine-isotope fractionations. *Geochim. Cosmochim. Acta* **67**, 3267–3281 (2003).

39. A. J. Magenheim, A. J. Spivack, P. J. Michael, J. M. Gieskes, Chlorine stable isotope composition of the oceanic crust: Implications for Earth's distribution of chlorine. *Earth Planet. Sci. Lett.* **131**, 427–432 (1995).

40. W. Wei, M. Kastner, A. Spivack, Chlorine stable isotopes and halogen concentrations in convergent margins with implications for the Cl isotopes cycle in the ocean. *Earth Planet. Sci. Lett.* **266**, 90–104 (2008).

41. I. Grevemeyer, H. Villinger, Gas hydrate stability and the assessment of heat flow through continental margins. *Geophys. J. Int.* **145**, 647–660 (2001).

42. H. M. Villinger, A. M. Tréhu, I. Grevemeyer, Seafloor marine heat flux measurements and estimation of heat flux from seismic observations of bottom simulating reflectors, in *Geophysical Characterization of Gas Hydrates*, M. Riedel, E. C. Willoughby, S. Chopra, Eds. (Geophysical Developments Series, Society of Exploration Geophysics, 2010), chap. 18, pp. 279–300.

43. A. M. Tréhu, D. S. Stakes, C. D. Bartlett, J. Chevallier, R. A. Duncan, S. K. Goffredi, S. M. Potter, K. A. Salamy, Seismic and seafloor evidence for free gas, gas hydrates, and fluid seeps on the transform margin offshore Cape Mendocino. *J. Geophys. Res.* **108**, 2263 (2003).

44. W. T. Wood, J. F. Gettrust, R. Chapman, G. D. Spence, R. D. Hyndman, Decreased stability of methane hydrates in marine sediments owing to phase-boundary roughness. *Nature* **420**, 656–660 (2002).

45. A. M. Tréhu, Subsurface temperatures beneath southern Hydrate Ridge, in *Proceedings of the Ocean Drilling Program, Scientific Results, 204*, A. M. Tréhu, G. Bohrmann, M. E. Torres, F. S. Colwell, Eds. (Ocean Drilling Program, 2006), pp. 1–26.

46. P. Linke, E. Suess, M. E. Torres, V. Martens, W. D. Rugh, W. Ziebis, L. D. Kulm, In situ measurement of fluid flow from cold seeps at active continental margins. *Deep Sea Res. Part I Oceanogr. Res. Pap.* **41**, 721–739 (1994).

47. M. E. Torres, J. McManus, D. E. Hammond, M. A. de Angelis, K. U. Heeschen, S. L. Colbert, M. D. Tryon, K. M. Brown, E. Suess, Fluid and chemical fluxes in and out of sediments hosting methane hydrate deposits on Hydrate Ridge, OR, I: Hydrological provinces. *Earth Planet. Sci. Lett.*, **201**, 525–540 (2002).

48. M. D. Tryon, K. M. Brown, M. E. Torres, Fluid and chemical flux in and out of sediments hosting methane hydrate deposits on Hydrate Ridge, OR, II: Hydrological processes. *Earth Planet. Sci. Lett.* **201**, 541–557 (2002).

49. M. Haeckel, E. Suess, K. Wallmann, D. Rickert, Rising methane gas bubbles form massive hydrate layers at the seafloor. *Geochim. Cosmochim. Acta* **68**, 4335–4345 (2004).

50. M. E. Torres, K. Wallmann, A. M. Tréhu, G. Bohrmann, W. S. Borowski, H. Tomaru, Gas hydrate growth, methane transport, and chloride enrichment at the southern summit of Hydrate Ridge, Cascadia margin off Oregon. *Earth Planet. Sci. Lett.* **266**, 225–241 (2004).

51. X. Liu, P. B. Flemings, Passing gas through the hydrate stability zone at southern Hydrate Ridge, offshore Oregon. *Earth Planet. Sci. Lett.* **241**, 211–226 (2006).

52. K. You, P. B. Flemings, A. Malinverno, T. S. Collett, K. Darnell, Mechanisms of methane hydrate formation in geological systems. *Rev. Geophys.* **57**, 1146–1196 (2019).

53. R. McCaffrey, A. I. Qamar, R. W. King, R. Wells, G. Khazaradze, C. A. Williams, C. W. Stevens, J. J. Vollick, P. C. ZwicK, Fault locking, block rotation and crustal deformation in the Pacific Northwest. *Geophys. J. Int.* **169**, 1315–1340 (2007).

54. C. F. You, P. R. Castillo, J. M. Gieskes, L. H. Chan, A. J. Spivack, Trace element behavior in hydrothermal experiments: Implications for fluid processes at shallow depths in subduction zones. *Earth Planet. Sci. Lett.* **140**, 41–52 (1996).

55. R. H. James, D. E. Allen, W. E. Seyfried Jr., An experimental study of alteration of oceanic crust and terrigenous sediments at moderate temperatures (51 to 350°C): Insights as to chemical processes in near-shore ridge-flank hydrothermal systems. *Geochim. Cosmochim. Acta* **67**, 681–691 (2003).

56. M. Underwood, M. E. Torres, Data report: Composition of clay minerals from hemipelagic sediments at Hydrate Ridge, Cascadia Subduction Zone, in *Proceedings of the Ocean Drilling Program, Scientific Results, 204*, A. M. Tréhu, G. Bohrmann, M. E. Torres, F. S. Colwell, Eds. (Ocean Drilling Program, 2006), pp. 1–15.

57. B. Carson, M. L. Holmes, K. Umstattd, J. C. Strasser, H. P. Johnson, Fluid expulsion from the Cascadia accretionary prism: Evidence from porosity distribution, direct measurements, and GLORIA imagery. *Philos. Trans. Royal Soc. A* **335**, 331–340 (1991).

58. M. E. MacKay, G. F. Moore, G. R. Cochrane, J. C. Moore, L. V. D. Kulm, Landward vergence and oblique structural trends in the Oregon margin accretionary prism: Implications and effect on fluid flow. *Earth Planet. Sci. Lett.* **109**, 477–491 (1992).

59. C. Goldfinger, L. V. D. Klum, R. S. Yeats, L. McNeill, C. Hummon, Oblique strike-slip faulting of the central Cascadia submarine forearc. *J. Geophys. Res.* **102**, 8217–8243 (1997).

60. M. E. Torres, B. M. A. Teichert, A. M. Tréhu, W. Borowski, H. Tomaru, Relationship of pore water freshening to accretionary processes in the Cascadia margin: Fluid sources and gas hydrate abundance. *Geophys. Res. Lett.* **31**, L22305 (2004).

61. B. M. A. Teichert, M. E. Torres, G. Bohrmann, A. Eisenhauer, Fluid sources, fluid pathways and diagenetic reactions across an accretionary prism revealed by Sr and B geochemistry. *Earth Planet. Sci. Lett.* **239**, 106–121 (2005).

62. M. E. MacKay, Structural variation and landward vergence at the toe of the Oregon accretionary prism. *Tectonics* **14**, 1309–1320 (1995).

63. S. Han, S. M. Carbotte, J. P. Canales, M. R. Nedimović, H. Carton, Along-trench structural variations of the subducting Juan de Fuca plate from multichannel seismic reflection imaging. *J. Geophys. Res. Sol. Ea.* **123**, 3122–3146 (2018).

64. C. Goldfinger, L. V. D. Kulm, R. S. Yeats, C. Hummon, G. J. Huftile, A. R. Niem, L. C. McNeill, Oblique strike-slip faulting of the Cascadia submarine forearc: The Daisy Bank fault zone off central Oregon, in *Subduction Top to Bottom: AGU Geophysical Monograph 96*, G. E. Bebout, D. Scholl, S. Kirby, J. P. Platt, Eds. (American Geophysical Union, 1996), pp. 65–74.

65. H. J. Tobin, J. C. Moore, M. E. MacKay, D. L. Orange, L. V. D. Klum, Fluid flow along a strike-slip fault at the toe of the Oregon accretionary prism: Implications for the geometry of frontal accretion. *Geol. Soc. Am. Bull.* **105**, 569–582 (1993).

66. B. Applegate, C. Goldfinger, M. E. MacKay, L. V. D. Klum, C. G. Fox, R. W. Embley, P. J. Meis, A left-lateral strike-slip fault seaward of the Oregon Convergent Margin. *Tectonics* **11**, 465–477 (1992).

67. C. Goldfinger, L. V. D. Klum, R. S. Yeates, B. Applegate, M. E. MacKay, G. F. Moore, Transverse structural trends along the Oregon convergent margin: Implications for Cascadia earthquake potential and crustal rotations. *Geology* **20**, 141–144 (1992).

68. A. T. Fisher, Permeability within basaltic oceanic crust. *Rev. Geophys.* **36**, 143–182 (1998).

69. H. J. Tobin, J. C. Moore, G. F. Moore, Fluid pressure in the frontal thrust of the Oregon accretionary prism: Experimental constraints. *Geology* **22**, 979–982 (1994).

70. J. S. Seewald, K. W. Doherty, T. R. Hammar, S. P. Liberatore, A new gas-tight isobaric sampler for hydrothermal fluids. *Deep Sea Res. Part I* **49**, 189–196 (2002).

71. A. Boetius, F. Wenzhöfer, Seafloor oxygen consumption fuelled by methane from cold seeps. *Nat. Geosci.* **6**, 725–734 (2013).

72. Shipboard Scientific Party, Site 1252, in *Proceedings of the Ocean Drilling Program, Initial Reports, 204*, A. M. Tréhu, G. Bohrmann, F. R. Rack, M. E. Torres, M. E., Eds. (Ocean Drilling Program, 2003), pp. 1–62.

73. A. L. Kurapov, N. A. Pelland, D. L. Rudnick, Seasonal and interannual variability in along-slope oceanic properties off the US West Coast: Inferences from a high-resolution regional model. *J. Geophys. Res. Ocean.* **122**, 5237–5259 (2017).

74. E. D. Sloan, *Clathrate Hydrates of Natural Gases* (Marcel Dekker, ed. 2, 1998).

75. R. D. Hyndman, K. Wang, T. Yuan, G. D. Spence, Tectonic sediment thickening, fluid expulsion, and the thermal regime of subduction zone accretionary prisms: The Cascadia margin off Vancouver Island. *J. Geophys. Res.* **98**, 21865–21876 (1993).

76. A. M. Tréhu, G. Lin, E. Maxwell, C. Goldfinger, A seismic reflection profile across the Cascadia Subduction Zone offshore central Oregon: New constraints on methane distribution and crustal structure. *J. Geophys. Res. Solid Earth* **100**, 15101–15116 (1995).

77. B. J. Phrampus, R. N. Harris, A. M. Tréhu, Heat flow bounds over the Cascadia margin derived from bottom simulating reflectors and implications for thermal models of subduction. *Geochem. Geophys. Geosyst.* **18**, 3309–3326 (2017).

78. M. S. Salmi, H. P. Johnson, R. N. Harris, Thermal environment of the Southern Washington region of the Cascadia subduction zone. *J. Geophys. Res. Solid Earth* **122**, 5852–5870 (2017).

79. N. Ganguly, G. D. Spence, N. R. Chapman, R. D. Hyndman, Heat flow variations from bottom simulating reflectors on the Cascadia margin. *Mar. Geol.* **164**, 53–68 (2000).

80. P. Tishchenko, C. Hensen, K. Wallmann, C. S. Wong, Calculation of the stability and solubility of methane hydrate in seawater. *Chem. Geol.* **219**, 37–52 (2005).

81. G. E. Claypool, A. V. Milkov, Y.-J. Lee, M. E. Torres, W. S. Borowski, H. Tomaru, Microbial methane generation and gas transport in shallow sediments of an accretionary

complex, southern Hydrate Ridge (ODP Leg 204), offshore Oregon, USA, in *Proceedings of the Ocean Drilling Program, Scientific Results, v. 204*, A. M. Tréhu, G. Bohrmann, M. E. Torres, F. S. Colwell, Eds. (Ocean Drilling Program, 2006), pp. 1–52.

82. W. B. F. Ryan, S. M. Carbotte, J. O. Coplan, S. O'Hara, A. Melkonian, R. Arko, R. A. Weissel, V. Ferrini, A. Goodwillie, F. Nitsche, J. Bonczkowski, R. Zemsky, Global multi-resolution topography synthesis. *Geochem. Geophys. Geosyst.* **10**, Q03014 (2009).

83. J. T Watt, D. S. Brothers, Systematic characterization of morphotectonic variability along the Cascadia convergent margin: Implications for shallow megathrust behavior and tsunami hazards. *Geosphere* **17**, 95–117 (2021).
